# Roles of the different components of magnesium chelatase in abscisic acid signal transduction

**DOI:** 10.1007/s11103-012-9965-3

**Published:** 2012-09-27

**Authors:** Shu-Yuan Du, Xiao-Feng Zhang, Zekuan Lu, Qi Xin, Zhen Wu, Tao Jiang, Yan Lu, Xiao-Fang Wang, Da-Peng Zhang

**Affiliations:** 1 MOE Systems Biology and Bioinformatics Laboratory, School of Life Sciences, Tsinghua University, Beijing, 100084 China; 2 College of Biological Sciences, China Agricultural University, Beijing, 100094 China; 3 State Key Laboratory of Integrated Management of Pest Insects and Rodents, Institute of Zoology, Chinese Academy of Sciences, Beijing, 100101 China

**Keywords:** H subunit of Mg-chelatase, I subunit of Mg-chelatase, D subunit of Mg-chelatase, GUN4, ABA binding, ABA signal transduction

## Abstract

The H subunit of Mg-chelatase (CHLH) was shown to regulate abscisic acid (ABA) signaling and the I subunit (CHLI) was also reported to modulate ABA signaling in guard cells. However, it remains essentially unknown whether and how the Mg-chelatase-catalyzed Mg-protoporphyrin IX-production differs from ABA signaling. Using a newly-developed surface plasmon resonance system, we showed that ABA binds to CHLH, but not to the other Mg-chelatase components/subunits CHLI, CHLD (D subunit) and GUN4. A new *rtl1* mutant allele of the *CHLH* gene in *Arabidopsis thaliana* showed ABA-insensitive phenotypes in both stomatal movement and seed germination. Upregulation of *CHLI1* resulted in ABA hypersensitivity in seed germination, while downregulation of *CHLI* conferred ABA insensitivity in stomatal response in *Arabidopsis*. We showed that CHLH and CHLI, but not CHLD, regulate stomatal sensitivity to ABA in tobacco (*Nicotiana benthamiana*). The overexpression lines of the *CHLD* gene showed wild-type ABA sensitivity in *Arabidopsis*. Both the *GUN4*-RNA interference and overexpression lines of *Arabidopsis* showed wild-type phenotypes in the major ABA responses. These findings provide clear evidence that the Mg-chelatase-catalyzed Mg-ProtoIX production is distinct from ABA signaling, giving information to understand the mechanism by which the two cellular processes differs at the molecular level.

**Electronic supplementary material:**

The online version of this article (doi:10.1007/s11103-012-9965-3) contains supplementary material, which is available to authorized users.

## Introduction

Abscisic acid (ABA) is an essential hormone to regulate plant growth and development and to control plant adaptation to environmental challenges (reviewed in Finkelstein and Rock 2002; Adie et al. 2007). As one of the highly complex plant cell signaling systems, ABA signaling begins with signal perception, which triggers downstream signaling cascades to induce the final physiological responses. It has been believed that the ABA signal is sensed by cells with multiple receptors including plasma membrane and intracellular receptors (Assmann 1994; Finkelstein et al. 2002; Verslues and Zhu 2007). In the past decades, ABA signal transduction has been extensively studied, and numerous signaling components, including ABA receptors, have been identified. These ABA signaling regulators involve diverse proteins, which localize to different cellular compartments including plasma membrane, cytosolic space and nucleus. Functioning on cell surface, two candidate plasma membrane ABA receptors—an unconventional G-protein-coupled receptor (GPCR) GCR2 and a novel class of GPCR-type G proteins GTG1 and GTG2—have been reported (Liu et al. 2007a, b; Johnston et al. 2007; Pandey et al. 2009), though it is controversial whether GCR2 regulates ABA-mediated inhibition of seed germination and post-germination growth (Gao et al. 2007; Guo et al. 2008). GTGs are positive regulators of ABA signaling and interacts with the sole *Arabidopsis* G protein α subunit GPA1 (Pandey and Assmann 2004), which may negatively regulate ABA signaling by inhibiting the activity of GTG-ABA binding (Pandey et al. 2009). Many other membrane-associated proteins, such as phospholipases C/D (Fan et al. 1997; Sanchez and Chua 2001; Zhang et al. 2004, 2009), other GPCR members (such as GCR1) and G proteins (Wang et al. 2001; Pandey and Assmann 2004; Pandey et al. 2006), and receptor-like kinases (Osakabe et al. 2005), have been reported to be involved in ABA signaling. However, whether these plasma membrane-localized proteins cooperates with plasma membrane ABA receptors to regulate early events of ABA signaling processes on the cell surface remains an interesting and open question.

Intracellular ABA signaling regulators involve numerous proteins of diverse identities such as various protein kinases, type-2C/A protein phosphatases (PP2C/A), ubiquitin E3 ligases involved in degradation of ABA signaling proteins, and various classes of transcription factors (for reviews, see Shinozaki et al. 2003; Fan et al. 2004; Seki et al. 2007; Cutler et al. 2010). Most recently, PYR/PYL/RCAR proteins, the members of a START domain superfamily, were reported to function as cytosolic ABA receptors by inhibiting directly type 2C protein phosphatases (Ma et al. 2009; Park et al. 2009; Santiago et al. 2009). A PYL/PYR/RCAR-mediated ABA signaling pathway from ABA perception to downstream gene expression has been reconstituted in vitro (Fujii et al. 2009; Cutler et al. 2010). In this PYR/PYL/RCAR-mediated ABA signaling pathway, PP2Cs relay ABA signal directly from the PYR/PYL/RCAR ABA receptors to their downstream regulators SNF1-related protein kinase 2s (SnRK2s), which activate an ABF/AREB/ABI5 clade of bZIP-domain transcription factors via a protein phosphorylation process, and finally induce physiological ABA responses (Fujii et al. 2009; Cutler et al. 2010). However, it is widely believed that the networks of ABA signaling pathways are highly complex, and connections of other numerous ABA signaling components with the PYR/PYL/RCAR ABA receptors remain to be explored.

We previously reported that the magnesium-protoporphyrin IX (Mg-ProtoIX) chelatase large subunit (Mg-chelatase H subunit CHLH/putative ABA receptor ABAR), a chloroplast/plastid protein, binds ABA and functions in ABA signaling, thus meeting the essential criteria of a candidate receptor for ABA in *Arabidopsis thaliana* (Shen et al. 2006; Wu et al. 2009). We further identified a CHLH-mediated ABA signaling pathway in which CHLH antagonizes a WRKY-domain transcription repressor to relieve ABA-responsive genes of inhibition (Shang et al. 2010). Although the identity of CHLH as an ABA receptor is controversial (Müller and Hansson 2009; Tsuzuki et al. 2011), we provide multiple lines of evidence to show that CHLH binds ABA (Shen et al. 2006; Wu et al. 2009; Wang et al. 2011) on the one hand, and on the other, consistent with our observations (Shen et al. 2006; Wu et al. 2009; Shang et al. 2010), evidence from independent groups reveals that CHLH mediates ABA signaling in guard cells of both *Arabidopsis* (Legnaioli et al. 2009; Tsuzuki et al. 2011) and peach (*Prunus persica*) leaves (Jia et al. 2011a). Also, it has been demonstrated that CHLH is a key component connecting the circadian clock with ABA-mediated plant drought responses in *Arabidopsis* (Legnaioli et al. 2009) and mediates ABA signaling in fruit ripening of both peach (Jia et al. 2011a) and strawberry (*Fragaria ananassa*, Jia et al. 2011b). These data consistently demonstrate that CHLH is an essential ABA signaling regulator in plant cells.

CHLH has multiple functions in plant cells. One of its functions is to chelate magnesium to protoporphyrin IX, which provides Mg-ProtoIX in the chlorophyll biosynthesis pathway (Gibson et al. 1996; Willows et al. 1996; Walker and Willows 1997; Guo et al. 1998; Papenbrock et al. 2000). The second role of CHLH is to mediate plastid-to-nucleus retrograde signaling, known as Genomes Uncoupled 5 (GUN5), and this function in the retrograde signaling may be connected with its role in catalyzing production of Mg-ProtoIX (Mochizuki et al. 2001; Nott et al. 2006). It has been well established that Mg-chelatase functions in catalyzing Mg-ProtoIX production as a hetero-tetramer, which is composed of Mg-chelatase subunits H, I (CHLI), D (CHLD) (Gibson et al. 1996; Willows et al. 1996; Walker and Willows 1997; Guo et al. 1998; Papenbrock et al. 2000) and a supplementary and essential component GUN4 (Genomes Uncoupled 4) that binds CHLH and activates Mg-chelatase (Larkin et al. 2003; Peter and Grimm 2009; Adhikari et al. 2011). A recent report showed that, besides CHLH, CHLI also mediates guard cell signaling in response to ABA (Tsuzuki et al. 2011). However, it remains essentially unknown whether Mg-chelatase heterotetramer complex or only two subunits CHLH and CHLI function in ABA signaling, and why the Mg-ProtoIX production process may differ from the CHLH-mediated ABA signaling. To explore this mechanism is of importance to understanding complex ABA signaling pathways. Here we report that, using a newly-developed surface plasmon resonance (SPR) technique, CHLH, but not CHLI, CHLD or GUN4, was shown to interact with ABA. Further findings demonstrate that CHLH and CHLI, but not CHLD nor GUN4, are ABA signaling regulators in the major ABA responses, and that the functions of CHLH and CHLI are not limited to ABA signaling in guard cells. The data provide clear and direct evidence that the Mg-chelatase-catalyzed Mg-ProtoIX production is distinct from ABA signaling, giving information to understand the mechanism by which the two cellular processes differs at the molecular level.

## Results

### Interactions of CHLH/ABAR with CHLI and CHLD

**Fig. 1:**
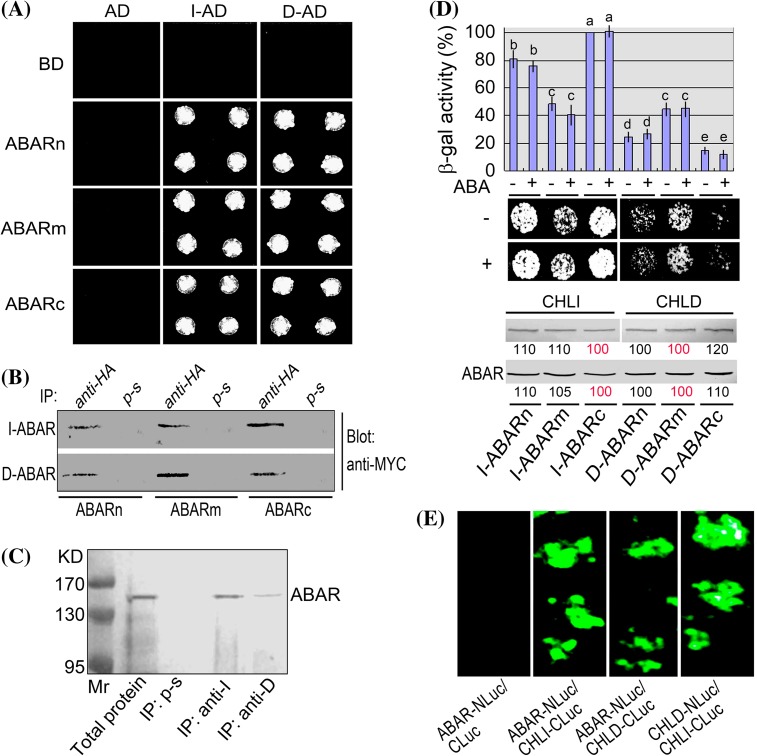
Interactions of CHLH/ABAR with CHLI1 and CHLD. **a** Test of yeast growth in SD medium lacking Leu, Trp, His, and Ade. AD, activation domain in the prey vector. BD, the DNA binding domain (BD) in the bait vector. ABARn, ABARm and ABARc indicate, respectively, N-terminal peptide (amino acid residues 1–691), median peptide (amino acid residues 347–1038) and C-terminal peptide (amino acid residues 692–1381) of CHLH/ABAR, which are linked, respectively, to BD in the bait vector. CHLI1 (indicated by I) and CHLD (indicated by D) are linked to the AD in the prey vector (indicated by I-AD, D-AD), respectively. The yeast cells were co-transformed with both vectors harboring CHLD (or CHLI1) and ABARs. The yeast cells co-transformed with the I-AD/D-AD of the prey vector and the bait vector carrying BD domain only (empty vector), or with the AD empty vector and BD-vector carrying ABARn, ABARm or ABARc, were taken as negative controls. **b** Coimmunoprecipitation assay in the same yeast cells as described above in (**a**). MYC-tagged ABARs are coimmunoprecipitated with HA-tagged CHLI1 or CHLD from yeast total proteins. Total proteins were extracted from the yeast cells transformed, respectively, with construct pairs I-ABARs and D-ABARs. Immunoprecipitation (IP) was performed with anti-HA serum or preimmune serum (p-s, as a negative control), and the immunoprecipitate was blotted (Blot) with the anti-MYC serum. The experiments were repeated three times with the same results. **c** ABAR and CHLI1 (or CHLD) are coimmunoprecipitated from Arabidopsis total proteins. Immunoprecipitation (IP) was performed with either the anti-CHLI1 (anti-I) or anti-CHLD (anti-D) serum and immunoblotting with the anti-ABAR serum. Immunoprecipitation with preimmune serum (p-s) was taken as a control. KD indicates the molecular mass. The experiments were repeated three times with the same results. **d** CHLH/ABAR interacts with CHLI1 more tightly than with CHLD. *Top panel* β-gal activity of the yeast cells harboring both ABARs and CHLI1 (I-ABARs) in comparison with that of the yeast cells expressing ABARs and CHLD (D-ABARs). β-Gal activity is presented as relative units (%), normalized relative to the highest activity of the *I*-*ABARc*-transformed cells. Each value is the mean ± SE of five independent biological determinations and different letters indicate significant differences at *P* < 0.05 (Duncan’s multiple range test). *Middle panel* drop test of yeast growth of the above-mentioned transformed yeast cells. Note that ABA does not affect these bimolecular interactions. ‘+’ indicates (±)ABA (2 μM) treatments, and ‘−’ ethanol solution (for solubilizing ABA) (as a control). *Bottom panel* levels of the CHLI, CHLD or ABAR protein in the transformed yeast cells. Before drop test, yeast cells of 10 mL with the same OD_600_ were collected and proteins were extracted for immunoblotting with ABAR/CHLH, CHLI, and CHLD antiserum. Relative band intensities, which are normalized relative to the intensity with the highest activity of the *I*-*ABARc*-transformed cells among the I-ABARs interactions, and to that with the highest activity of the *D*-*ABARm*-transformed cells among the D-ABARs interactions (indicated by red 100), are indicated by numbers in boxes below the bands. **e** Firefly Luc complementation imaging to test protein–protein interactions. The tobacco leaves were transformed by infiltration using a needleless syringe with construct pairs ABAR-N-terminal half of Luc (NLuc)/CHLI1-C-terminal half of Luc (CLuc), ABAR-NLuc/CHLD-CLuc, CHLD-NLuc/CHLI1-CLuc or ABAR-NLuc/CLuc (as a negative control, see *left panel*)

It is of importance to elucidate clearly the interactions between CHLH and CHLI or CHLD to understand the mechanisms of both Mg-chelatase function and CHLH/ABAR-mediate ABA signaling. Previous reports showed that CHLH is a magnesium- and protoporphyrin IX-binding protein and interacts directly with CHLD (Grafe et al. 1999; Masuda 2008) and GUN4 (Larkin et al. 2003), but whether it interacts directly with CHLI remains unclear. We assayed interactions of different truncated CHLH/ABAR proteins with CHLD and CHLI1. CHLI includes two isoforms in *Arabidopsis*, CHLI1 (encoded by At4g18480 locus) and CHLI2 (encoded by At5g45930 locus), of which CHLI1 is a major isoform (Huang and Li 2009). The two CHLI isoforms function redundantly (Rissler et al. 2002; Kobayashi et al. 2008; Huang and Li 2009). The assayed truncated CHLH/ABAR proteins include the C-terminal half (ABARc), N-terminal half (ABARn) and middle region (ABARm) of CHLH/ABAR, which correspond to the amino acid residues 692–1381, 1–691, and 347–1038, respectively. In the yeast two-hybrid system, ABARn, ABARm and ABARc are linked, respectively, to the DNA binding domain (BD) in the bait vector; CHLI1 and CHLD are linked to the activation domain (AD) in the prey vector (indicated by I-AD, D-AD), respectively. The yeast cells were co-transformed with both vectors harboring CHLD (or CHLI1) and ABARs. The yeast cells co-transformed with the I-AD/D-AD of the prey vector and the empty bait vector carrying BD domain only, or with the AD empty vector and BD-vector carrying ABARn, ABARm, ABARc or empty BD vector, were taken as negative controls. The results showed that all these truncated CHLH/ABAR proteins interact with both CHLD and CHLI1 in the yeast two-hybrid system (Fig. 1a, b, d). The different negative controls showed no interaction signal (Fig. 1a), indicating that these detected bimolecular interactions are specific and reliable. It is noteworthy that CHLH/ABAR is a trans-chloroplast-membrane protein (Shang et al. 2010), and thus the truncated CHLH/ABAR could not move into the yeast nucleus to interact with CHLD and CHLI1 if the truncated CHLH/ABAR are associated with yeast membranes in this GAL4-based two hybrid system that requires interactions in the nucleus. One possible explanation for the interaction of the truncated CHLH/ABAR with CHLD and CHLI1 in the yeast cells is that CHLH/ABAR has a low hydrophobicity (Shang et al. 2010) and may likely not linked to the membranes of yeast cells that lacks plastids, and another possibility is that the truncation-caused mutations prevent association of the truncated proteins into yeast membranes. Nevertheless, we further tested these bimolecular interactions in plant systems. Coimmunoprecipitation assay in plant total protein and an in vivo luciferase complementation imaging assay (LCI) confirmed that CHLH interacts with both CHLD and CHLI1 (Fig. 1c, e). Interestingly, we found that CHLH/ABAR interacts more strongly with CHLI1 than with CHLD, and that the C-terminal half of CHLH/ABAR protein (ABARc) interacts most strongly with CHLI1 in comparison with the ABARn and ABARm truncated proteins, as evidenced by both β-galactosidase activity and drop test of yeast growth in the yeast two-hybrid system (Fig. 1d). We carefully performed these yeast-two hybrid assays and showed that the differences in the detected bimolecular-interaction intensities were not caused by the differences in the expression levels of the related proteins, in which the CHLH protein showed substantially no differences in their amounts among different treatments, and the quantities of CHLI and CHLD proteins were also carefully controlled in order that their amounts in the stronger bimolecular interactions were not higher than those in the weaker interactions (Fig. 1d), indicating that the estimations of the bimolecular-interaction intensities are reliable. None of the interactions between ABARs and CHLI1 or CHLD was affected by ABA treatment in the yeast two-hybrid system (Fig. 1d).

### CHLH, but not CHLI, CHLD or GUN4, binds ABA

**Fig. 2:**
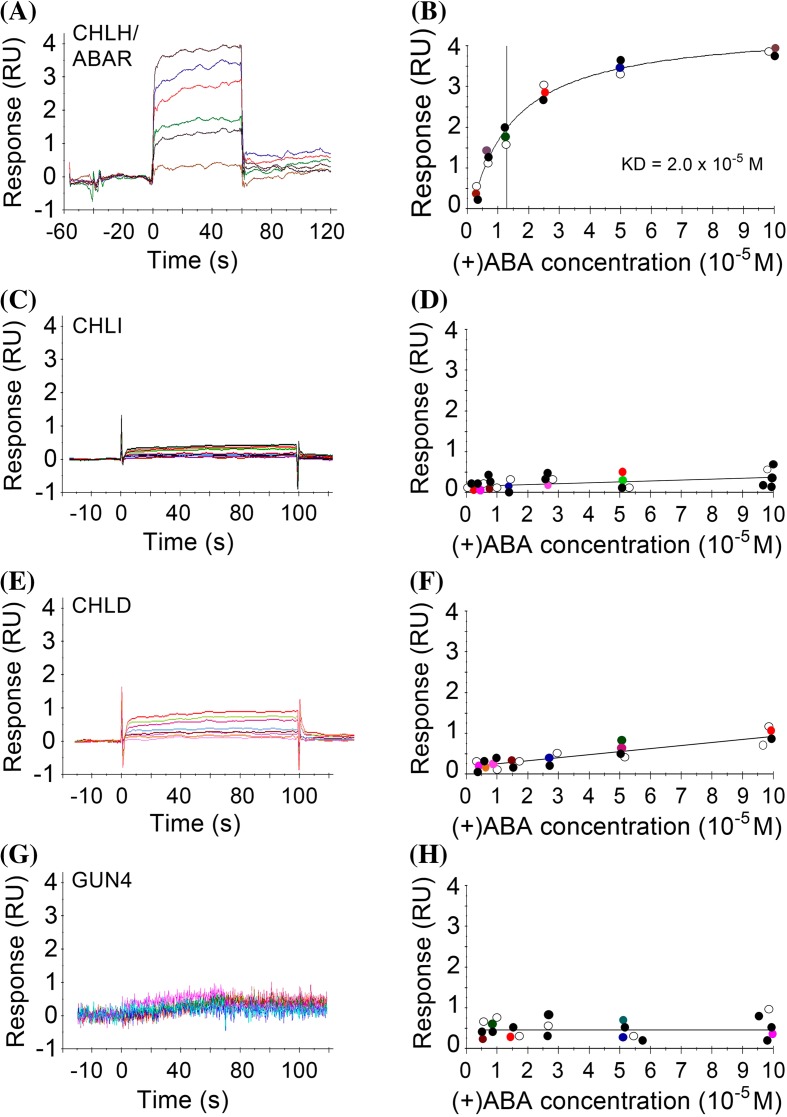
SPR assays: CHLH, but not CHLI1, CHLD or GUN4, binds ABA. The sample proteins [CHLH (**a**) and (**b**); CHLI (**c**) and (**d**); CHLD (**e**) and (**f**); GUN4 (**g**) and (**h**)] were immobilized to a chip by an amino-coupling process, and (+)ABA binding to these proteins was tested by recording the response data. *Left panels* (**a, c, e, g**) show the row data of a representative response record, and *right panels* (**b, d, f, h**) show the corresponding saturation curves of ABA binding to each of the proteins where the *colour circles* indicate the data presented in the corresponding *left panels*, while the *filled circles* and *open circles* represent the data, respectively, from other two independent repetitions. The experiments were repeated independently five times with the similar results

It is essential to investigate ABA-binding abilities of all the four components of Mg-chelatase to understand their possible roles in ABA signaling. We newly adopted the surface plasmon resonance (SPR) technique for assaying ABA binding for these Mg-chelatase proteins. We observed that only CHLH binds ABA with a saturation curve typical for receptor-ligand binding (Fig. 2a, b). However, it should be noted that, in this SPR system, the detected ABA-binding affinity of CHLH was low (equilibrium dissociation constant Kd = 20 μM), which may be due to the technical limitations of this technique for testing interaction of this huge, more or less hydrophobic CHLH protein (about 150 kDa) with a small ligand.

In contrast to CHLH, CHLI (Fig. 1c. d), CHLD (Fig. 2e, f) or GUN4 (Fig. 2g, h) did not show substantial ABA-binding abilities. These three proteins showed only a low and nonspecific ABA-binding background (Fig. 2d, f, h), which may serve as a negative control.

### New observations of a mutant allele of *CHLH* gene, *rtl1*

**Fig. 3:**
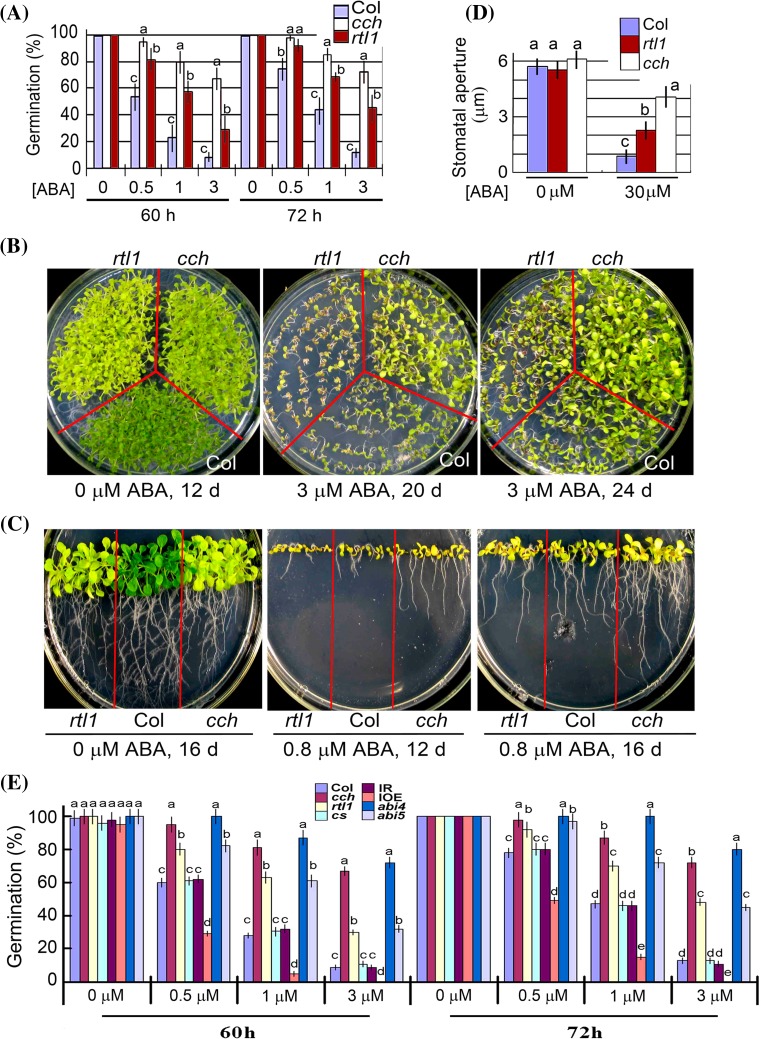
New phenotypes of *rtl1*, a new mutant allele of *CHLH* gene. **a** The *rtl1* mutant seeds showed ABA insensitivity in germination like *cch* mutant. Seed germination rate of the wild-type plants (Col) and two mutant alleles *cch* and *rtl1* of *CHLH* gene in the ABA-free medium (0 μM ABA) and ABA-containing medium (0.5, 1 and 3 μM) from 60 to 72 h after stratification. Each value is the mean ± SE of five independent biological determinations and different letters indicate significant differences at *P* < 0.05 (Duncan’s multiple range test) when comparing values within the same ABA concentration. **b** and **c** The *rtl1* mutant seedlings showed wild-type ABA sensitivity, while the *cch* mutant seedlings showed ABA insensitivity in early growth. The wild-type plants (Col) and *cch* and *rtl1* mutant seeds were directly planted in the ABA-free medium (0 μM ABA) and ABA-containing medium [3 μM in (**b**) and 0.8 μM in (**c**)], and seedling growth was recorded 12 d, 16 d or 24 d after stratification as indicated. **d** Both *cch* and *rtl1* mutants showed ABA insensitivity in ABA-induced stomatal closure. Each value is the mean ± SE of five independent biological determinations and different letters indicate significant differences at *P* < 0.05 (Duncan’s multiple range test) when comparing values within the same group (within the same ABA concentration applied for assaying stomatal aperture); n = 60 apertures per experiment. **e** Comparison of ABA-induced inhibition of seed germination among different genotypes. Seed germination rate of the wild-type plants (Col), *cch*, *rtl1*, cs, *abi4* (*abi4*-*1*), *abi5* (*abi5*-*1*), a *CHLI*-RNAi (IR) and a *CHLI*-overexpression lines in the ABA-free medium (0 μM ABA) and ABA-containing medium (0.5, 1 and 3 μM) from 60 to 72 h after stratification. Each value is the mean ± SE of five independent biological determinations and different letters indicate significant differences at *P* < 0.05 (Duncan’s multiple range test) when comparing values within the same ABA concentration

A previous report identified a new mutant allele of *CHLH* gene *rtl1* (*r*apid *t*ranscription of *l*eaves*1*) with a single nucleotide substitution, resulting in a single amino acid mutation Leu690 → Phe (see Supplementary Fig. 1), which is susceptible to dehydration with defect in stomatal response to ABA, but the mutant was shown to have wild-type response in ABA-induced inhibition of seed germination and early developmental arrest (Tsuzuki et al. 2011). We assayed the ABA responses of this mutant in our experimental conditions, and observed that the seed germination of the *rtl1* mutant was significantly insensitive to ABA with a weaker phenotype than *cch* mutant (Fig. 3a), which is a mutant harbors a single nucleotide substitution at a different site of *CHLH* gene, resulting in a single amino acid mutation Pro642 → Leu (Mochizuki et al. 2001; see Supplementary Fig. 1) and leading to ABA-insensitive phenotypes in seed germination, early seedling growth and stomatal movement (Shen et al. 2006; Wu et al. 2009). This observation of the defect of the *rtl1* mutant in ABA response of seed germination is inconsistent with the previous report (Tsuzuki et al. 2011). However, we observed that the *rtl1* mutant has wild-type response to ABA in early seedling growth (Fig. 3b, c), which is consistent with the previous observation (Tsuzuki et al. 2011), while *cch* mutant showed significant ABA-insensitive phenotype in early seedling growth (Fig. 3b, c) as we previously reported (Shen et al. 2006; Wu et al. 2009). Also, we confirmed the previous observation (Tsuzuki et al. 2011) that the *rtl1* mutant is insensitive to ABA in stomatal movement, and found that the ABA-insensitive phenotype of this mutant in stomatal movement was significantly weaker that the *cch* mutant (Fig. 3d).

### Upregulation of CHLI1 confers ABA hypersensitivity in seed germination, while downregulation of CHLI results in ABA insensitivity in stomatal response

**Fig. 4:**
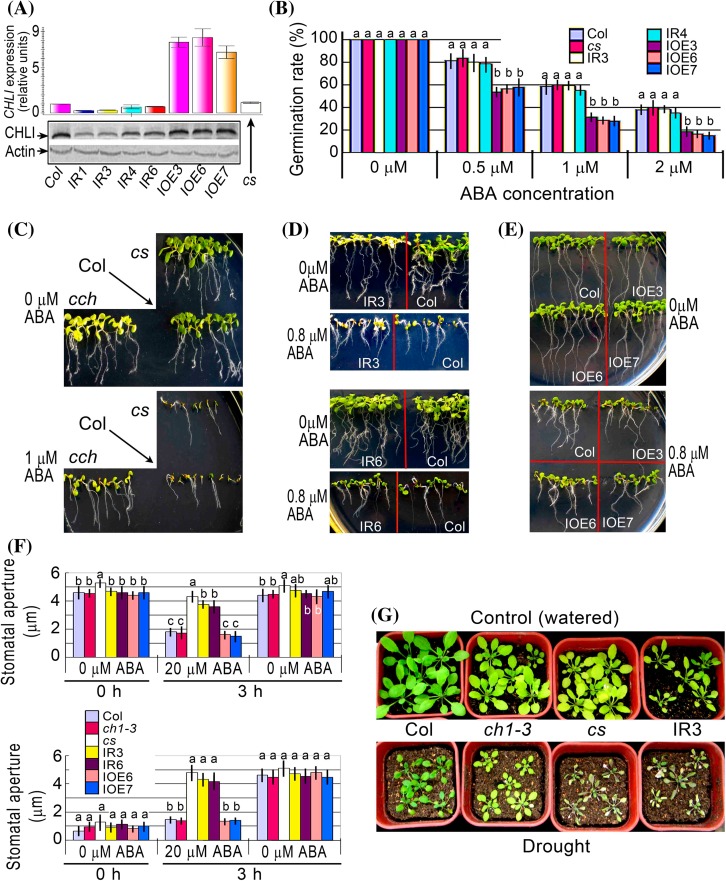
Phenotypes of the overexpression and RNAi lines of *CHLI* gene. **a** Test of the expression levels of *CHLI* (including *CHLI1* and *CHLI2*) gene in the transgenic lines. *Top panel* real-time PCR data. *Bottom panel* immunoblotting data with Actin as a loading control. Col, wild-type Col-0 plants; IR1, IR3, IR4 and IR6, *CHLI*-RNAi lines 1, 3, 4 and 6, respectively; IOE3, IOE6 and IOE7, *CHLI1*-overexpression lines 3, 6 and 7, respectively; *cs*, a T-DNA insertion line of the *CHLI1* gene. **b** Seed germination rate of the wild-type plants (Col) and different transgenic lines as described in (**a**) in the ABA-free medium (0 μM ABA) and ABA-containing medium (0.5, 1 and 2 μM) 60 h after stratification. Each value is the mean ± SE of five independent biological determinations and *different letters* indicate significant differences at *P* < 0.05 (Duncan’s multiple range test) when comparing values within the same ABA concentration. **c–e**, Early seedling growth of the wild-type plants (Col), *cch* and *cs* mutants and different transgenic lines [IR3 and IR6, (**d**); IOE3, IOE6 and IOE7, (**e**)] as described in (**a**) in the ABA-free medium (0 μM ABA) and ABA-containing medium [0.8 μM, (**d**) and (**e**); 1 μM, (**c**)] 12 d after stratification. The experiments were repeated independently five times with the same results. **f** ABA-induced stomatal closure (*top panel*) and ABA-inhibited stomatal opening (*bottom panel*) for wild-type Col, *ch1*-*3* and *cs* mutants and different transgenic lines (IR3, IR6, IOE6 and IOE7) as described in (**a**) in the ABA-free medium (0 μM ABA) and ABA-containing medium (20 μM). Values are the mean ± SE from three independent experiments and different letters indicate significant differences at *P* < 0.05 (Duncan’s multiple range test) when comparing values within the same ABA concentration. n = 60 apertures per experiment. **g** Whole-plant status in the water loss assays. Intact plants were well watered (Control) or drought stressed (Drought) by withholding water for 15 d for assaying water loss of the two mutants, *ch1*-*3* and *cs*, and a CHLI-RNAi line IR3 in comparison with wild-type Col. The entire experiment was replicated three times with similar results

Previous report showed that downregulation of CHLI resulted in ABA insensitive phenotype in stomatal movement, but did not affect ABA responses in seed germination and early seedling growth (Tsuzuki et al. 2011). Because the authors used a knockout mutant of *CHLI1* (SAIL_230_D11), which exhibited a severe dwarf and pale-green phenotype (Tsuzuki et al. 2011), and therefore may interfere with the investigations related to ABA responses, we verified these observations with a T-DNA insertion knockdown mutant in *CHLI1* gene, known as *cs* (Mochizuki et al. 2001), and transgenic overexpression and RNAi lines of *CHLI1* gene. We used the conserved sequences to created *CHLI*-RNAi lines, which targeted to both *CHLI1* and *CHLI2*. We identified the overexpression and RNAi lines (Fig. 4a) using the antibody against CHLI1, which recognized CHLI1 and CHLI2. The *cs* mutant and the RNAi lines showed wild-type response to ABA in seed germination and early seedling growth (Fig. 4b–d; Supplementary Fig. 2a, b), but ABA insensitivity in stomatal response and reduced tolerance to dehydration (Fig. 4f, g), which is essentially consistent with previous observations (Tsuzuki et al. 2011). Interestingly, however, the overexpression lines of *CHLI1* gene showed significantly hypersensitive to ABA in ABA-induced inhibition of seed germination (Fig. 4b) with wild-type phenotypes in ABA-induced early seedling growth arrest (Fig. 4e; Supplementary Fig. 2c) and in stomatal response to ABA (Fig. 4f). A chlorophyll-deficient mutant *ch1*-*3*, which contain lesions in the gene encoding chlorophyll a oxygenase (Espineda et al. 1999), was used as a control and showed wild-type ABA response in stomatal movement and drought tolerance (Fig. 4f, g), indicating that CHLI-mediated stomatal response to ABA is independent of chlorophyll biosynthesis.

We compared the mutant *cs* and the *CHLI*-RNAi and *CHLI*-overexpression lines with *cch*, *rtl1* and two well characterized mutants *abi4*-*1* and *abi5*-*1* in their ABA sensitivity in seed germination. While confirming ABA hypersensitivity of the *CHLI*-overexpression line and wild-type ABA response of the *cs* mutant and the *CHLI*-RNAi line, we observed that *cch* mutant displayed substantially the same intensity of ABA-insensitivity as *abi4*-*1* mutant at low ABA concentrations (<1 μM), but weaker ABA-insensitivity than *abi4*-*1* mutant at high ABA concentrations (1 to 3 μM) (Fig. 3e). The *cch* mutant showed higher degree of ABA-insensitivity than *abi*-*5* mutant, and *rtl1* mutant displayed substantially the same intensity of ABA-insensitivity as *abi5*-*1* mutant and weaker ABA-insensitivity than *abi4*-*1* mutant (Fig. 3e).

### Downregulation of *CHLH* or *CHLI*, but not *CHLD*, reduces stomatal sensitivity to ABA in tobacco, and upregulation of CHLD has no impact on ABA sensitivity in *Arabidopsis*

**Fig. 5:**
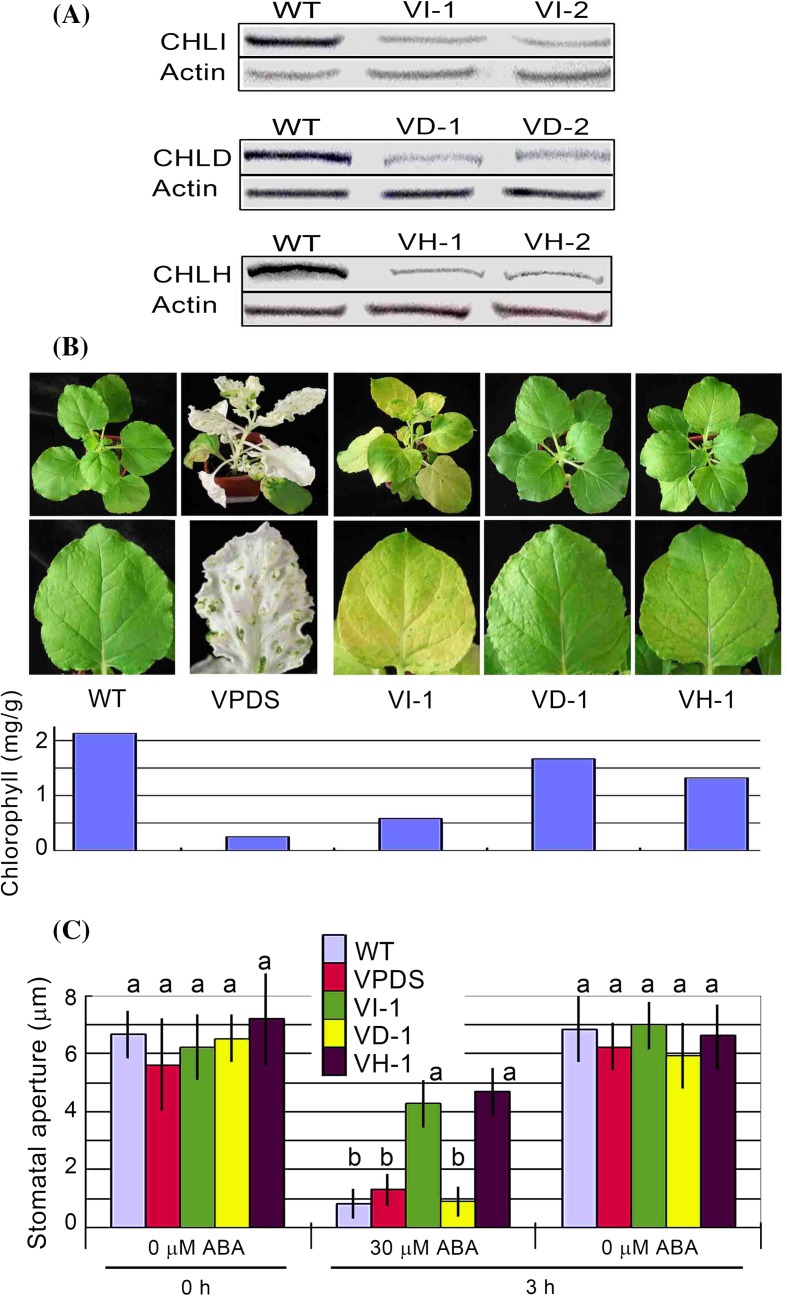
ABA-induced stomatal closure of the RNAi lines for *CHLH*, *CHLI* and *CHLD* genes in tobacco plants. **a** Immunoblotting analysis for CHLI (including CHLI1 and CHLI2, *top*) in the *CHLI*-VIGS lines VI-1 and VI-2, CHLD (*middle*) in the *CHLD*-VIGS lines VD-1 and VD-2, and CHLH proteins (*bottom*) in the *CHLH*-VIGS lines VH-1 and VH-2. The immunoblotting signals in the wild-type non-transgenic lines (WT) were serviced as controls, and Actin was used as a loading control. **b** Plant status of the wild-type tobacco (WT) and the VI-1, VD-1 and VH-1 transgenic lines with a VIGS line for *PDS* (phytoene desaturase) gene (VPDS) as a control. *Bottom panel* shows the chlorophyll concentrations in the corresponding lines. **c** ABA-induced stomatal closure for the wild-type plants (WT) and different transgenic lines (VPDS, VI-1, VD-1 and VH-1) as described in (**a**) and (**b**) in the ABA-free medium (0 μM ABA) and ABA-containing medium (30 μM). Values are the mean ± SE from three independent experiments and different letters indicate significant differences at *P* < 0.05 (Duncan’s multiple range test) when comparing values within the same ABA concentration. n = 60 apertures per experiment

We failed to obtain the *CHLD*-RNAi lines through the transgenic manipulations as described in the Materials and Methods section because all the transgenic lines were pale and not able to establish growth. The T-DNA insertion knockout mutants of the *CHLD* gene available in the public resources such as Arabidopsis Biological Resource Center (ABRC) are lethal (Strand et al. 2003; Tsuzuki et al. 2011). Given that the CHLH, CHLI and CHLD are highly conserved in both their sequences and functions from bacteria to high plants (Gibson et al. 1996; Willows et al. 1996; Walker and Willows 1997; Guo et al. 1998; Papenbrock et al. 2000; for some plant CHLH, CHLI and CHLD, see Supplementary Figs. 3-6), we designed RNAi-constructs according to the *Arabidopsis* *CHLH, CHLI* and *CHLD* genes, and used a *tobacco rattle virus* (TRV) based virus induced gene silencing (VIGS) system (Liu et al. 2002) to down-regulate the expression of *CHLD* in tobacco (*N. benthamiana*). The gene silencing efficiency of the VIGS system was assessed by suppressing the expression of the phytoene desaturase (*PDS*) gene in tobacco, in which silencing of *PDS* leads to the inhibition of carotenoid synthesis, causing the plants to a photo-bleached phenotype (Fig. 5b, VPDS) as described previously (Liu et al. 2002; Kumagai et al. 1995). We successfully obtained more than ten lines that expressed the *CHLD* gene at relatively low levels, had slightly yellow leaves and survived well (Fig. 5a, b). Also, we created the VIGS lines for *CHLH* and *CHLI* genes both to serve as controls and to verify the functions of these two genes in tobacco. We observed that the transgenic tobacco leaves displayed pale or even blanched phenotypes when the amount of any of the three subunit proteins was downregulated to a very low level, verifying their indispensible role for chlorophyll biosynthesis as functional Mg-chelatase subunits (data not shown). We showed that the VIGS lines of *CHLD* gene showed wild-type stomatal response to ABA, while the VIGS lines of both the *CHLH* and *CHLI* genes showed ABA insensitive phenotype in ABA-induced stomatal closure (Fig. 5c; Supplementary Fig. 7). These data indicate that CHLH and CHLI regulate ABA response in stomatal movement in both *Arabidopsis* and tobacco plants, and that CHLD does not function in this stomatal response to ABA.

**Fig. 6:**
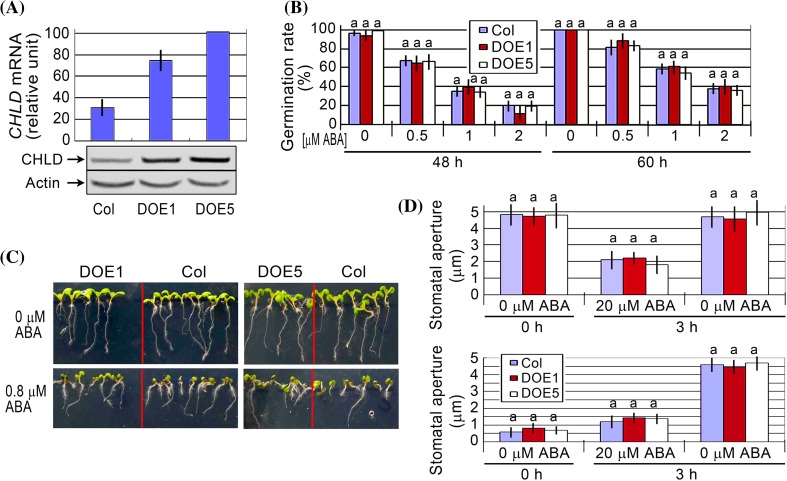
Phenotypic analysis of the *CHLD*-overexpression lines. **a** Test of the expression levels of *CHLD* gene in the wild-type Col plants and the transgenic lines DOE1 and DOE5 by quantitative PCR analysis (*top*) and immunoblotting assays (*bottom*, with Actin as a loading control). **b** Seed germination rate of the wild-type plants (Col) and two transgenic lines DOE1 and DOE5 in the ABA-free medium (0 μM ABA) and ABA-containing medium (0.5, 1 and 2 μM) 48 and 60 h after stratification. Each value is the mean ± SE of five independent biological determinations and different letters indicate significant differences at *P* < 0.05 (Duncan’s multiple range test) when comparing values within the same ABA concentration. **c** Early seedling growth of the wild-type plants (Col) and two transgenic lines DOE1 and DOE5 in the ABA-free medium (0 μM ABA) and ABA-containing medium (0.8 μM) 12 d after stratification. The experiments were repeated independently five times with the same results. **d** ABA-induced stomatal closure (*top panel*) and ABA-inhibited stomatal opening (*bottom panel*) for the wild-type plants (Col) and two transgenic lines DOE1 and DOE5 in the ABA-free medium (0 μM ABA) and ABA-containing medium (20 μM). Values are the mean ± SE from three independent experiments and *different letters* indicate significant differences at *P* < 0.05 (Duncan’s multiple range test) when comparing values within the same ABA concentration. n = 60 apertures per experiment

We further created the overexpression lines of *CHLD* gene in *Arabidopsis* (Fig. 6a), and observed that these overexpression lines showed wild-type phenotypes in ABA-induced inhibition of seed germination (Fig. 6b) and early seedling growth arrest (Fig. 6c; Supplementary Fig. 2d), and ABA-induced stomatal closure and inhibition of stomatal opening (Fig. 6d). These data are essentially consistent with the data mentioned above in the VIGS lines in tobacco, indicating that CHLD is not involved in ABA signaling.

### Down- or up-regulation of *GUN4* gene does not alter ABA response

**Fig. 7:**
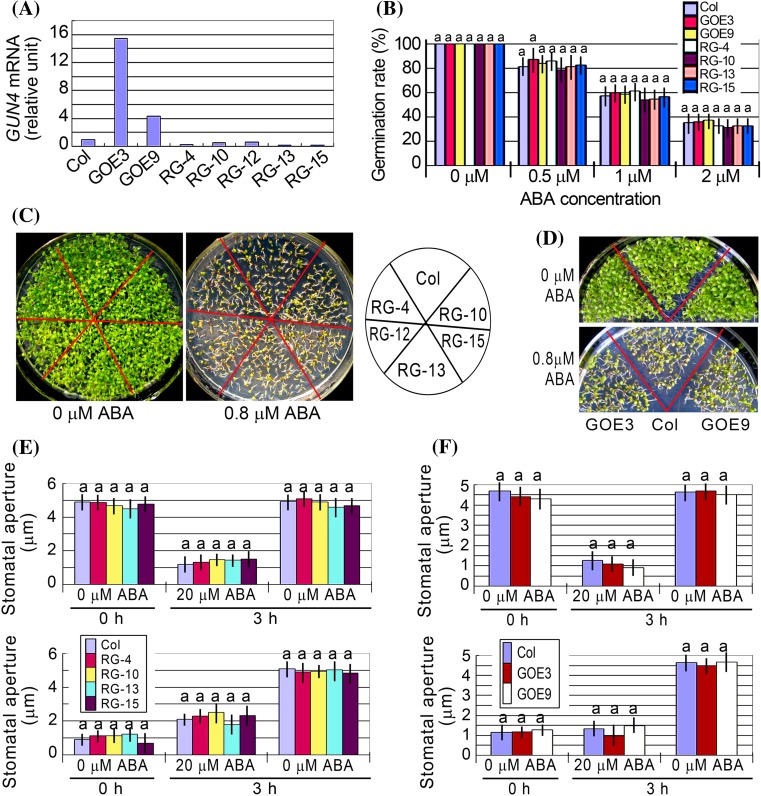
Phenotypes of the overexpression and RNAi lines of *GUN4* gene. **a** Test of the expression levels of *GUN4* gene in the transgenic lines by real-time PCR analysis. Col, wild-type Col-0 plants; GOE3 and GOE9, *GUN4*-overexpression lines 3 and 9, respectively; RG4, RG10, RG12, RG13 and RG15, *GUN4*-RNAi lines 4, 10, 12, 13 and 15, respectively. **b** Seed germination rate of the wild-type plants (Col) and different transgenic lines as described in (**a**) in the ABA-free medium (0 μM ABA) and ABA-containing medium (0.5, 1 and 2 μM) 60 h after stratification. Each value is the mean ± SE of five independent biological determinations and different letters indicate significant differences at *P* < 0.05 (Duncan’s multiple range test) when comparing values within the same ABA concentration. **c** and **d**, Early seedling growth of the wild-type plants (Col), different *GUN4*-RNAi lines (**c**) and two *GUN4*-overexpression lines (**d**) in the ABA-free medium (0 μM ABA) and ABA-containing medium (0.8 μM) 12 d after stratification. The experiments were repeated independently five times with the same results. (**e**) and (**f**), ABA-induced stomatal closure (top panel) and ABA-inhibited stomatal opening (bottom panel) for wild-type Col and different *GUN4*-RNAi lines (**e**) and two *GUN4*-overexpression lines (**f**) in the ABA-free medium (0 μM ABA) and ABA-containing medium (20 μM). Values are the mean ± SE from three independent experiments and different letters indicate significant differences at *P* < 0.05 (Duncan’s multiple range test) when comparing values within the same ABA concentration. n = 60 apertures per experiment

We created the *GUN4*-RNAi and overexpression lines (Fig. 7a), and observed that all these transgenic RNAi and overexpression lines showed wild-type phenotypes in ABA-induced inhibition of seed germination (Fig. 7b) and early seedling growth arrest (Fig. 7c, d; Supplementary Fig. 2e, f), and ABA-induced stomatal closure and inhibition of stomatal opening (Fig. 7e, f). These data demonstrate that GUN4 is not involved in ABA signaling.

## Discussion

### CHLH and CHLI are positive regulators of ABA signaling: new observations in *Arabidopsis* and tobacco

We previously showed that the *cch* mutation of *CHLH* gene reduced ABA sensitivity in all the three major ABA responses including ABA-induced inhibition of seed germination, post-germination growth arrest, and promotion of stomatal closure and inhibition of stomatal opening (Shen et al. 2006; Wu et al. 2009). The data in the present experiment (Figs. 3, 4c) are consistent with these previous observations. However, a new mutant of *CHLH* gene *rtl1*, allelic to *cch*, was reported to have the substantial same ABA-related phenotypes as *cch*, which reduced only stomatal sensitivity to ABA but did not affect other two major ABA response during seed germination and post-germination growth (Tsuzuki et al. 2011). In our present experiment, however, we showed that the *rtl1* mutation reduces significantly ABA sensitivity both in seed germination and in stomatal response, though the ABA response in post-germination growth was not affected (Fig. 4), supporting the idea that the functions of CHLH are not limited to mediating stomatal signaling in response to ABA.

We observed that this inconsistency in the phenotypes in seed germination and seedling growth of the *cch* (and also *rtl1*) mutant is mainly due to variations in growth conditions. The *cch* mutant was identified as a light- and drought-sensitive mutant (Mochizuki et al. 2001; Shen et al. 2006). We previously emphasized that the growth conditions with a sufficient irrigation, high relative air humidity (60–70 %), moderate light intensity (about 120 μmol photons m^−2^ s^−1^) to ensure the good growth status of the *cch* mutant parental plants are of critical importance to their progeny to display the ABA-related phenotypes (Wu et al. 2009). Otherwise, the stressed mutant parental plants had progeny with shrunken seeds, whose insensitive phenotypes to ABA became weaker in germination and post-germination growth, but the strong ABA-insensitive phenotype in stomatal movement was not changed (Wu et al. 2009).

We reproduced well the significant ABA insensitive phenotypes of the *cch* mutant in all the three major ABA responses with plump seeds of quality harvested from the mutant plants grown in the above-mentioned favourable conditions (Shen et al. 2006; Wu et al. 2009; Shang et al. 2010; and this experiment). This is also true for the *rtl1* mutant. The phenomenon of the conditional ABA insensitivity in seed germination and post-germination growth may be likely due to a stress-induced upregulation of ABA-responsive mechanisms independent of CHLH-mediated signaling in the *cch* (or *rtl1*) mutant (Wu et al. 2009), which needs to be clarified in the future.

The similar phenomenon of the conditional ABA-insensitive phenotypes in seed germination and post-germination growth was also observed in the *CHLH*-RNAi plants. We previously observed that the ABA insensitivity in stomatal response has a significant negative correlation with the CHLH levels in the RNAi lines (Shen et al. 2006). However, whereas a globally negative correlation of the ABA insensitivity with the CHLH levels was found in germination and post-germination growth, the phenotypes become weaker and dependent on good growth conditions when the CHLH levels are low to a certain extent especially when the RNAi plants show yellow leaves (Shen et al. 2006, see Supplementary Fig. 3 in this reference). We observed that the CHLH-RNAi plants showed strong ABA insensitive phenotypes in all the three ABA responses when the *CHLH* expression was reduced to an extent at which the residual CHLH protein levels were higher than 30 %, which could ensure that the RNAi plants remained green (Shen et al. 2006, see Fig. 2 and Supplementary Fig. 3 in this reference). These findings are essentially consistent with the above-mentioned observations in the *cch* or *rtl1* mutants both with yellow leaves.

All the missense mutants of *CHLH* gene, including *abar*-*2* (Wu et al. 2009), *abar*-*3* (Wu et al. 2009), *cch* (Shen et al. 2006; Wu et al. 2009) and *rtl1* (Tsuzuki et al. 2011) but except for *gun5* (Mochizuki et al. 2001; Shen et al. 2006), show altered ABA sensitivity in the major ABA responses (Supplementary Fig. 1). Additionally and noteworthily, several independent groups provided evidence that CHLH mediates stomatal signaling in response to ABA in *Arabidopsis* (Legnaioli et al. 2009; Tsuzuki et al. 2011) as well as in peach (*P. persica*) (Jia et al. 2011a). In the present experiment, we showed that CHLH also regulates stomatal response to ABA in tobacco (Fig. 5). Interestingly, CHLH functions in the regulation ABA signaling in fleshy fruit ripening such as peach (Jia et al. 2011a) and strawberry (*F. ananassa*, Jia et al., 2011b). All these findings support the idea that CHLH is a conserved ABA signaling regulator in plant cells.

It is interesting that, previously, CHLI was shown to be involved in stomatal response to ABA in *Arabidopsis* (Tsuzuki et al. 2011). In the present experiment, we confirmed the role of the CHLI in stomatal response to ABA in *Arabidopsis* as well as in tobacco (Figs. 4, 5), and observed that down regulation of CHLI did not affect ABA responses in seed germination and seedling growth, while upregulation of CHLI1 enhanced ABA sensitivity in ABA-induced inhibition of seed germination (Fig. 4b), suggesting that CHLI likely regulates ABA signaling in both stomatal movement and seed germination. This is possible because the *CHLI* gene has two copies in *Arabidopsis* genome and double knockout mutants of two *CHLI* genes are lethal (Huang and Li 2009), while the leaky mutants (including the RNAi lines) may not show ABA-related phenotypes in seed germination and seedling growth.

Taken together, all the data demonstrate that CHLH and CHLI are two positive regulators of ABA signaling, which may be likely to cooperate to function in plant cell response to ABA.

### CHLH, but not CHLI, CHLD or GUN4, is an ABA-binding protein

CHLH was originally isolated as an ABA-binding protein from total proteins of broad bean leaves by an ABA-affinity column that specifically binds CHLH via ABA, and was shown potentially to function in broad bean stomatal signaling in response to ABA (Zhang et al. 2002), though the ABA-affinity technique was questioned because ABA was immobilized on the affinity resin at its carboxyl group that was shown to be required for ABA’s function (Cutler et al. 2010). However, the ABA binding ability of CHLH was confirmed by both the same ABA-affinity system (Wu et al. 2009) and a ^3^H-labeled ABA binding assay (Shen et al. 2006; Wu et al. 2009). Further, we showed that the C-terminal half of CHLH is essential both for ABA binding and for ABA signaling that was evidenced by expression of this C-terminal protein in wild-type and *cch* mutant plants (Wu et al. 2009).

In contrast to the PYR/PYL/RCAR receptor for ABA, a cytosolic protein with a low molecular mass (about 20 kDa) and a highly hydrophilic nature (Ma et al. 2009; Park et al. 2009; Santiago et al. 2009), the CHLH protein is a chloroplast-membrane protein (Shang et al. 2010), and has a high molecular mass (about 150 kDa) and a slightly-hydrophobic nature. We observed that the CHLH protein aggregates and becomes rapidly unstable in solution (data not shown), which makes ABA binding assay difficult (Wang et al. 2011) and may likely be a reason why ABA binding of CHLH was not detected by other groups with the ^3^H-labeled ABA binding assay (Müller and Hansson 2009; Tsuzuki et al. 2011). Therefore, we adopted a new approach to test ABA binding of CHLH protein so that the data may be easily reproducible, which includes a new system to produce highly active CHLH protein with an insect cell line, and a surface plasmon resonance (SPR) system (Biacore T200, GE). The results showed that CHLH binds ABA with a saturation curve typical for receptor-ligand binding, while other Mg-chelatase components/subunits CHLI, CHLD and GUN4 do not bind ABA (Fig. 2). These data, easily reproduced with the insect-cell-produced protein and the SPR equipment, confirmed qualitatively the ABA-binding nature of the CHLH protein, and verified our previous observations (Shen et al. 2006; Wu et al. 2009).

However, the SPR system has technical limitations. In this system, CHLH protein should be linked to a sensor chip with carboxyl groups on its surface to which the sample protein is covalently immobilized via -NH_2_ bond of the protein, which may induce conformational change of the sample protein and reduce its activity. A structurally complex protein may partly lose its activity in some in vitro system, which was also observed in the ABA binding assay of the plasma membrane GTG1/2 receptors for ABA (Pandey et al. 2009). In addition, there is also a limitation of sensitivity to the weak signal for the signal-detecting system of the SPR equipment to test the interaction of this huge, hydrophobic CHLH protein with a small ligand ABA. The detected low ABA-affinity of CHLH protein is likely to be attributed to these technical limitations, though we previously detected high ABA affinity of this protein in a ^3^H-labeled ABA binding assay (Shen et al. 2006; Wu et al. 2009).

The CHLI subunit functions in ABA signaling in stomatal movement (Figs. 4, 5; and Tsuzuki et al. 2011) and also likely in seed germination (Fig. 4), while it does not directly interact with ABA (Fig. 2), suggesting that CHLI may function through interaction with CHLH. We showed that CHLI interacts directly with CHLH with stronger interaction intensity than that between CHLH and CHLD, which seems to support that CHLH interacts with CHLI to form a hetero-dimer to function in ABA signaling. We were not able to test whether CHLI affects the ABA binding activity of CHLH to be involved in ABA signaling because of the technical difficulties with the SPR system, but we could propose another possibility that CHLI may possibly modify the interactions between CHLH and its downstream regulators such as the WRKY40 transcription factor (Shang et al. 2010) to regulate ABA signaling, which needs studies in the future.

### CHLH and CHLI-mediated ABA signaling is distinct from Mg-protoporphyrin IX production

Previous experiments suggested that the CHLH-mediated signaling may be distinct from Mg-ProtoIX production and chloroplast retrograde signaling. No correlation between the Mg-ProtoIX levels and stomatal response to ABA was found (Shen et al. 2006), and both the *gun5* and *cch* mutations of *CHLH/ABAR/GUN5* gene reduced Mg-ProtoIX production and affected chloroplast retrograde signaling (Mochizuki et al. 2001), while the *cch* mutant alone, but not *gun5* mutant, showed ABA insensitive phenotypes (Shen et al. 2006; Wu et al. 2009). In the present experiment, we showed that, in contrast to CHLH and CHLI that are two positive regulators in ABA signaling, other two Mg-chelatase components CHLD and GUN4 are not involved in ABA signaling, as evidenced by *CHLD*-VIGS lines in tobacco (Fig. 5), *CHLD*-overexpression lines in *Arabidopsis* (Fig. 6), and *GUN4*-RNAi and overexpression lines in *Arabidopsis* (Fig. 7). Given that, as two components/subunits of Mg-chelatase, both CHLD and GUN4 are essential to the Mg-chelatase activity and Mg-ProtoIX production (Gibson et al. 1996; Larkin et al. 2003; Peter and Grimm 2009; Adhikari et al. 2011), the present data provide clear and direct evidence that the Mg-chelatase-catalyzed Mg-ProtoIX production is independent of the CHLH and CHLI-mediated ABA signaling, and give information to understand the mechanism by which the two cellular processes differs at the molecular level. Additionally, previous reports showed that CHLD (Strand et al. 2003), but not CHLI (Mochizuki et al. 2001), functions in chloroplast retrograde signaling, which contrasts with our above-mentioned findings that CHLI functions, but CHLD is not involved, in ABA signaling (Figs. 5, 6, 7), supporting the notion that the ProtoIX production and chloroplast retrograde signaling are independent of the CHLH and CHLI-mediated ABA signaling.

**Fig. 8:**
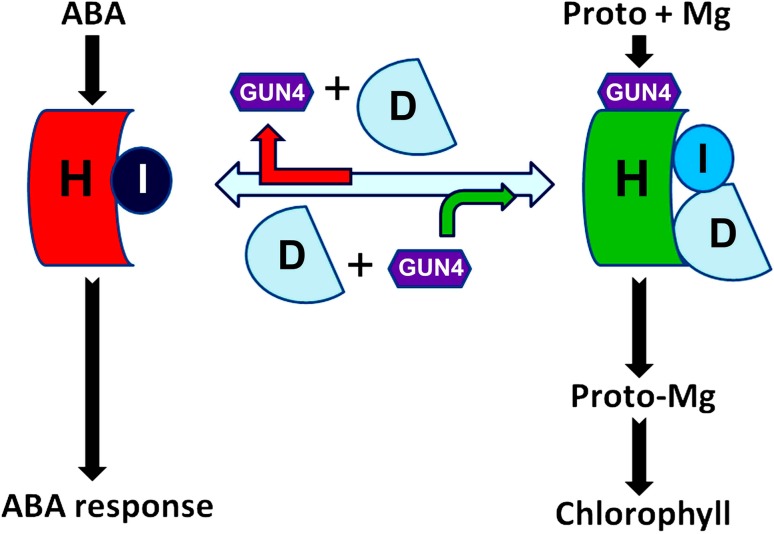
A model for distinction between CHLH- and CHLI-mediated ABA signaling and magnesium (Mg) chelating to protoporphyrin IX (Proto) catalyzed by a CHLH-CHLI-CHLD-GUN4 hetero-tetramer complex. See text for detailed explanation

Taking all the observations together, we propose a working model to explain the difference between the CHLH/CHLI-mediated ABA signaling and function of Mg-chelatase (Fig. 8). In this model, CHLH interacts with CHLI to form a hetero-dimer, which cooperates to regulate ABA signaling, while the function of Mg-chelatase requires all the four components/subunits CHLH, CHLI, CHLD and GUN4 to form a hetero-tetramer complex, which catalyzes magnesium chelating to protoporphyrin IX to produce Mg-ProtoIX. There may exist an equilibrium between the hetero-dimer and hetero-tetramer to meet the needs of two distinct functions, but the chlorophyll biosynthesis may be a privileged process, given that, a low level of the CHLH protein, which could downregulate ABA signaling, may not significantly reduce the chlorophyll contents in leaves (Shen et al. 2006).

## Materials and methods

### Plant materials, generation of transgenic lines and growth conditions

*Arabidopsis thaliana* ecotype Columbia (Col-0) was used in the generation of transgenic plants. The mutated *abi5* gene in the *abi5*-*1* mutant (ABRC stock number CS8105; named *abi5* in this report) was transferred from its background Wassilewskija (Ws) ecotype into Col-0 ecotype by backcrossing. To generate RNAi lines with down-regulated expression of *CHLI1* gene (Arabidopsis genomic locus At4g18480), *CHLD* (At1g08520) and *GUN4* (At3g59400), we chose a gene-specific fragment from each of their cDNAs. A 268-bp fragment corresponding to the region of nt 35–302 of the *CHLI1* cDNA, a 183-bp fragment corresponding to the region of nt 1334 to1516 of the *CHLD* cDNA, and a 337-bp fragment corresponding to the region of nt 429–765 of the *GUN4* cDNA, were amplified by PCR, respectively, with forward primer 5′-GCGTCGACAACTTCATCTCATCTTGCCCTAC-3′ and reverse primer 5′-CCATCGATTCTGGGCTTCTCCTTCACTCTC-3′ for *CHLI1* gene, forward primer 5′-CCCAAGCTTAAATGAGCAGCAACAGGAC-3′ and reverse primer 5′-CCATCGATTTGGAAGCATTGGCTTTAT-3′ for *CHLD* gene, and forward primer 5′-CCATCGATTGGTAGATTCGGATACAGCGTG-3′ and reverse primer 5′-GCGTCGACGTCTGCTCCTACTCCTGCCTG-3′ for *GUN4* gene. These fragments were inserted in sense orientation, respectively, into the pSK-int vector with the *Cla*I*/Sal*I sites for *CHLI1* gene, *Hind*III/*Cla*I sites for *CHLD* gene, and *Cla*I/*Sal*I sites for *GUN4* gene. The same fragments as above mentioned for each of three genes were amplified, respectively, with forward primer 5′-CGGAATTCTCTGGGCTTCTCCTTCACTCTC-3′ and reverse primer 5′-GGACTAGTAACTTCATCTCATCTTGCCCTAC-3′ for *CHLI1* gene, forward primer 5′-CGGAATTCAAATGAGCAGCAACAGGAC-3′ and reverse primer 5′-GGACTAGTTTGGAAGCATTGGCTTTAT-3′ for *CHLD* gene, and forward primer 5′-TCCCCCGGGTGGTAGATTCGGATACAGCGTG-3′ and reverse primer 5′-GGACTAGTGTCTGCTCCTACTCCTGCCTG-3′ for *GUN4* gene. These fragments were subsequently placed in antisense orientation, respectively, into the pSK-int vector already carrying the corresponding sense fragment with the *Eco*RI/*Spe*I sites for *CHLI1* gene, *Eco*RI/*Spe*I sites for *CHLD* gene, and *Sma*I/*Spe*I sites for *GUN4* gene. The entire RNAi cassette comprising the sense and antisense fragments interspersed by the *Actin II* intron was excised from the pSK-int using the flanking *Sac*I/*Apa*I sites and inserted into the *Sac*I/*Apa*I site of pSUPER1300(+) vector, yielding the *CHLI1* RNAi, *CHLD* RNAi and *GUN4* RNAi construct, respectively. The pSUPER1300(+) Super Promoter is a hybrid promoter combining a triple repeat of the *Agrobacterium tumefaciens* octopine synthase (ocs) activator sequences along with the mannopine synthase (mas) activator elements fused to the mas promoter, termed (Aocs)3AmasPmas (Ni et al. 1995). It is noteworthy that the *CHLI1* RNAi construct can target to both *CHLI1* and *CHLI2* (At5g45930) gene transcripts. The RNAi construct for each of the three genes was introduced into *Agrobacterium tumefaciens* GV3101 and transformed into Col-0 by floral dip method (Clough and Bent 1998). Transgenic plants were grown on Murashige–Skoog (MS) agar plates containing hygromycin (40 μg/ml) in order to screen the positive seedlings.

To create transgenic plant lines over-expressing *CHLI1*, *CHLD* and *GUN4* genes, the open reading frames (ORF) for these genes flanked by *Sma*I and *Sal*I sites were isolated by PCR, using the following primers: forward primer 5′-CCCCCGGGATGGCGTCTCTTCTTGGAACATC-3′ and reverse primer 5′-GCGTCGACTCAGCTGAAAATCTCGGCGAA C-3′ for *CHLI1*; forward primer 5′-CCCCCGGGATGGCGATGACTCCGGTCGC -3′ and reverse primer 5′-ACTCAAGAATTCTTCAGATCAGATAG -3′ for *CHLD*; forward primer 5′-CCCCCGGGATGGCGACCACAAACTCTC-3′ and reverse primer 5′-GCGTCGACTCAGAAGCTGTAATTTGTTT-3′ for *GUN4*. These ORFs cloned into pCAMBIA-1300-221 vector harboring a 35S promoter. Transgenic manipulation was done as previously described (Wu et al. 2009). The homologous T3 generation seeds or plants were used for analysis. At least ten transgenic lines were obtained for each of the constructs.

Plants were grown in a growth chamber at 20–21 °C on MS medium at about 80 μmol photons m^−2^ s^−1^, or in compost soil at about 120 μmol photons m^−2^ s^−1^ over a 16-h photoperiod. The *cs (cs1*-*1)* and *cch* mutants were generous gifts from Dr. J. Chory (The Salk Institute, La Jolla, CA). The *rtl1* mutant was a gift from Dr. T. Kinoshita (Nagoya University, Japan). The seed of *ch1*-*3* mutant (CS3362) was obtained from the Arabidopsis Biological Resource Center.

### Antibody production and immunoblotting

For the production of the antibody against CHLI and CHLD, the fragments corresponding to the cDNA of these genes were amplified and inserted into the *EcoR*I and *Xhol*I sites of pGEX4T-1 vector (Novagen). A 715-bp fragment of the *CHLI* cDNA was isolated using forward primer 5′-CCGGAATTC CCGGTTTATCCATTTGCAGCT-3′ and reverse primer 5′-CCGCTCGAGACTATCGAAACGAGCTCTCT-3′, which corresponds a common piece of amino-acid sequence of both CHLI1 and CHLI2. A 654-bp fragment of the *CHLD* cDNA was isolated using forward primer 5′-CCGGAATTCTTCTCAGAAGATAGAGGACGC-3′ and reverse primer 5′-CCGCTCGAGCTTCAGATCAGATAGTGCATC-3′. The GST-tagged fusion proteins were expressed in *Escherichia coli* BL21 (DE3). The affinity-purified fusion protein was used for standard immunization protocols in rabbit. The antisera were produced and tested for specificity as described previously (Wu et al. 2009). The extraction of Arabidopsis total protein, sodium dodecyl sulfate–polyacrylamide gel electrophoresis (SDS-PAGE) and immunoblotting were done essentially according to the previously described procedures (Shen et al. 2006; Wu et al. 2009).

### Quantitative real-time PCR

Real-time PCR for expression of various genes was performed as previously described (Wu et al. 2009) essentially according to the instructions provided for the BioRad Real-Time System CFX96TM C1000 Thermal Cycler (Singapore). The used primers were: forward primer 5′-GGTAACATTGTGCTCAGTGGTGG-3′ and reverse primer 5′-AACGACCTTAATCTTCATGCTGC-3′ for *Actin*; forward primer 5′-CGATGTTCCTTACCTTGTGGCAG-3′ and reverse primer 5′-CACGACCAGCGAAAACGATTG-3′ for *CHLH*; forward primer 5′-GACGGTTAGAGATGCTGATTTAC-3′ and reverse primer 5′-TCACTATGTCTCCTCTCAACCC-3′ for *CHLI* plus *CHLI2*; forward primer 5′-AAGTGGCAGTATGGCATTGAA-3′ and reverse primer 5′-AACCACCACCACAAGGAAGTC-3′ for *CHLD*; forward primer 5′-GGCGACCACAAACTCTCTCCACC-3′ and reverse primer 5′-GTTTCGGCAGTTGTGGCGGAG-3′ for *GUN4*.

### Expression and purification of CHLH, CHLI, CHLD and GUN4 proteins in the sf9 insect cell line

To construct *ABAR/CHLH*, *CHLI*, *CHLD* and *GUN4* expression vectors, the ORFs of these genes flanked by *Sal*I and *Kpn*I sites were cloned into pFastBac™ HFT-B (Invitrogen, CA), a kind of baculo-virus transfer vector. The sf9 cells (Invitrogen, about 1 × 10^9^) were infected with viruses expressing the Flag-tagged fusion proteins, respectively. The infected cells were seeded in flasks and cultured at 28 °C for 3 days. Cells were harvested and washed with a TBS buffer (50 mM Tris–HCl, pH 7.5, and 150 mM NaCl). Cell pellets were then lysed with sonication in the lysis buffer consisting of 50 mM Tris–HCl, pH 7.5, 150 mM NaCl, 5 mM 2-mercaptoethanol, 0.2 mg/ml trypsin inhibitor and 10 μg/ml leupeptin. After centrifugation at 17,000 g for 30 min, the supernatant was incubated with anti-FLAG M2 affinity resin (Sigma) at 4 °C for 2 h. The resin suspension was then washed with a wash buffer (10 mM, pH 7.5, 150 mM NaCl, 2 μg/ml leupeptin, and 5 mM 2-mercaptoethanol). Proteins bounding to anti-FLAG agarose, were eluted with 0.1 mM FLAG peptide (Asp Tyr Lys Asp Asp AspAsp Lys) in the wash buffer, purified by gel filtration and concentrated to 0.5–1 mg/ml by ultrafiltration.

### SPR assay

Surface plasmon resonance (SPR) measurements were performed using a Biacore T200 (GE Healthcare) equipped with a certified CM5 sensor chip with carboxyl groups on its surface. The sample proteins (>90 % pure based on Size Exclusion Chromatography) were covalently immobilized to saturate the surface of sensor chip via -NH_2_ bond using amino-coupling kit from Biacore. The surface of flow cell 2 was activated for 7 min with a 1:1 mixture of 0.1 M N-Hydroxysuccinimide (NHS) and 0.1 M 1-ethyl-3-(3-dimethylaminopropyl) carbodiimide hydrochloride (EDC) at a flow rate of 10 μl/min. The sample protein was immobilized to a density that saturates the surface at a concentration of 50 μg/ml in 10 mM sodium acetate (for CHLH and GUN4 at pH 4.5, for CHLI and CHLD at pH 4.0); flow cell 1 was left blank to serve as a reference surface. The surface was then blocked with a 7 min injection of 1 M ethanolamine, pH 8.0. To collect kinetic and affinity binding data, the analyte (+)-ABA in the HBS-EP running buffer (10 mM HEPES, 150 mM NaCl, 30 mM ethylene diamine tetraacetic acid (EDTA), and 0.005 % [v/v] surfactant P20, pH 7.4) was injected over flow cell 1 and flow cell 2 at concentrations of 6 to 100 μM at a flow rate of 30 ul/min and at 25 °C. The complex was allowed to associate and dissociate for 60 s, respectively. Data were collected and globally fitted to steady-state model available within Biacore Evaluation software v1.01.

### Analysis of protein interaction by yeast two-hybrid system and co-immunoprecipitation (CoIP) in yeast and in planta

Interaction between two proteins was assayed by a yeast Gal4-based two-hybrid system as described by the manufacturer (Clontech). The primers used for cloning the related cDNAs were as follows: for *ABAR*_*692*–*1381*_ (encoding C-terminal amino acid residues 692–1381 or ABARc): forward primer 5′-GGAATTCGGGAACATTCCCAATG-3′ and reverse primer 5′-ACGCGTCGACTTATCGATCGATCCCTTCGATC-3′; for *ABAR*_*1*–*691*_ (encoding N-terminal amino acid residues 1–691 or ABARn): forward primer 5′-CCGGAATTCATGGCTTCGCTTGTGTATTCTCC-3′ and reverse primer 5′-ACGCGTCGACGATAAGACTGTCGGGAAAAC-3′; for *ABAR*_*347*–*1038*_ (encoding median amino acid residues 347–1038 or ABARm): forward primer 5′-CCGGAATTCGCTTGAGGCTAGAGGTGCTA-3′ and reverse primer 5′-ACGCGTCGACGATGTTGTCAGTTCCCCAAA-3′; for the full length *CHLI1*: forward primer 5′-CGGAATTCATGGCGTCTCTTCTTGGAACATC-3′ and reverse primer 5′-ACCTCGAGCTCAGCTGAAAATCTCGGCGAA-3′; and for the full length *CHLD*: forward primer 5′-ACTGGATCCATATGGCGATGACTC-3′ and reverse primer 5′-ACGCTCGAGCTCAAGAATTCTTCAGATCAGATAG-3′. The cDNAs encoding the truncated *ABAR*s were inserted into the pGBKT7 plasmid by the *EcoR*I (5′ end) and *Sal*I (3′ end) sites to generate bait plasmids, and the cDNAs encoding *CHLI1* and *CHLD* were cloned into *EcoR*I (5′ end)/*Xho*I (3′ end) sites and *BamH*I (5′ end)/*Xho*I (3′ end) sites of pGADT7 plasmid to generate prey plasmids, respectively. The liquid β-galactosidase assays, including measurement of β-galactosidase activity, were performed according to the manufacturer’s protocol (Clontech) by using ONPG (o-nitrophenyl-β-d-galactopyranoside; Sigma Cat No. N-1127) as substrate, which is hydrolyzed to o-nitrophenol and D-galactose.

CoIP assays were performed in the extracts of both yeast cells and Arabidopsis plants. Yeast strains were grown using SD medium deficient in Leu, Trp, His and Ade to OD600 1.0 at 30 °C. Total proteins were prepared from yeast cells with an extraction buffer (2 mL/g cells) containing 50 mM HEPES (pH 7.4), 10 % glycerol (v/v), 1 mM EDTA, 0.1 % Triton X-100 (v/v), 100 μM PMSF, and 1 μg/mL each of aprotinin, leupeptin, and pepstatin A. The antibodies used were: mouse antibody (Medical and Biological Laboratories CO., LTD) specific to MYC-tagged truncated ABAR protein, and mouse antibody specific to HA- (hemagglutinin peptide epitope, Medical and Biological Laboratories CO., LTD) tagged CHLI1 and CHLD protein. Immunoprecipitation experiments were performed with protein A/G Plus-agarose beads (Santa Cruz), following the manufacturer’s protocol. In brief, cell lysates were pre-cleared with the protein A/G Plus-agarose beads and incubated with the anti-HA serum and the protein A/G Plus-agarose beads at 4 °C overnight in the extraction buffer. The beads were washed twice extensively with buffer A [50 mM Tris pH 8.0, 150 mM NaCl, 0.1 % Triton X-100 (v/v)] and buffer B [50 mM Tris pH 8.0, 0.1 % Triton X-100 (v/v)], respectively, and then resuspended in SDS-PAGE sample buffer. The immuno-precipitates were separated on a 10 % SDS-PAGE, analyzed by immunoblotting with anti-MYC serum.

For immunoprecipitation in Arabidopsis extracts, the total proteins (6 mg) were resuspended in the yeast protein extraction buffer (1 mL) as described above. The immunoprecipitation was done with the same procedures as described above except that the anti-ABAR and anti-CHLI1/anti-CHLD serum was used instead of the anti-MYC and anti-HA serum, and the beads were washed with the extraction buffer instead of the buffer A and buffer B.

### Test of protein–protein interaction by luciferase complementation imaging (LCI)

To further confirm the results of protein–protein interaction, we used a luciferase complementation imaging system according to previously described procedures (Shang et al. 2010) in which the firefly luciferase (Luc) enzyme is divided into the N- (NLuc) and C-terminal (CLuc) halves that do not spontaneously reassemble and function. Luc activity occurs only when the two fused proteins interact, resulting in reconstituted Luc enzyme. The primers used for cloning the related cDNAs were as follows: for *ABAR*-*NLuc*: forward primer 5′-GGGGTACCATGGCTTCGCTTGTGT-3′ and reverse primer 5′-ACGCGTCGACTCGATCGATCCCTTC-3′; for *CLuc*-*ABAR*: forward primer 5′-GGGGTACCATGGCTTCGCTTGTGT-3′ and reverse primer 5′-ACGCGTCGACTTATCGATCGATCCCTTC-3′; for *CLuc*-*CHLI1*: forward primer 5′-CGGGGTACCATGGCGTCTCTTCTTGGAACATC-3′ and reverse primer 5′-GCGTCGACTCAGCTGAAAATCTCGGCGAA-3′; for *CHLI1*-*NLuc:* forward primer 5′-CGGGGTACCATGGCGTCTCTTCTTGGAACATC-3′ and reverse primer 5′-GCGTCGACGCTGAAAATCTCGGCGAA-3′; for *CLuc*-*CHLD*: forward primer 5′-CGGGGTACCATGGCGATGACTCCGGTCGC-3′ and reverse primer 5′-GCGTCGACTCAAGAATTCTTCAGATCAG-3′; and for *CHLD*—*Nluc*: forward primer 5′-CGGGGTACCATGGCGATGACTCCGGTCGC-3′ and reverse primer 5′-GCGTCGAC AGAATTCTTCAGATCAGATA-3′.

The constructs were cloned into pCAMBIA-NLuc and pCAMBIA-CLuc at the *Kpn*I and *Sal*I sites. The constructs were mobilized into *A. tumefaciens strain* GV3101. Bacterial suspensions were infiltrated into young but fully expanded leaves of the 7-week old *N. benthamiana* plants using a needleless syringe. It is noteworthy that the amounts of the constructs were the same among treatments and controls for each group of assay. After infiltration, plants were grown first under dark for 12 h and then with 16 h light/d for 60 h at room temperature and the Luc activity were observed with a CCD imaging apparatus (Andor iXon, Andor, UK).

### VIGS assay and tobacco stomata aperture assay

We used a *tobacco rattle virus* (TRV) based virus induced gene silencing (VIGS) system (Liu et al. 2002) to down-regulate the expression of *CHLH*, *CHLI* and *CHLD* in tobacco. The VIGS assay was performed essentially according to previously described procedures (Liu et al. 2002). The primers used for cloning the related cDNAs were as follows: for *ABAR:* forward primer 5′-CCGGAATTCGGGAACATTCCCAATG-3′ and reverse primer 5′-CCGCTCGAG TTATCGATCGATCCCTTCGATC-3′; for *CHLI:* forward primer 5′-CCGGAATTCCCGGTTTATCCATTTGCAGCT-3′ and reverse primer 5′-CCGCTCGAG CCAACAAACCAGGCTCAAAGG-3′; and for *CHLD:* forward primer 5′-CCGGAATTCCGAGAAAAAGTCACAATCGATG-3′ and reverse primer 5′-CCGCTCGAGCGCCCTGCCAGCTTTCCCC-3′.

The fragments corresponding to the cDNAs of these genes were cloned into the *EcoR*I and *Xhol*I sites of pTRV2 vector. The constructs were mobilized into *A. tumefaciens strain* GV3101. *Agrobacterium* containing pTRV1 and pTRV2 were mixed in 1 : 1 ratio and infiltrated into the lower leafs of 4-leaf stage *Nicotiana benthamiana* plants using a needleless syringe. Each silencing experiment was repeated at least 3 times and each experiment included at least five independent plants. We assessed the gene silencing efficiency by suppressing the expression of the phytoene desaturase (*PDS*) gene in *N. benthamiana*. A mixture of *Agrobacterium* culture containing the pTRV2-PDS and pTRV1 was infiltrated as described above. About 7 days after infiltration, the upper leaves of the plant exhibited the silencing effect. Silencing of *PDS* leads to the inhibition of carotenoid synthesis, causing the plants to a photo-bleached phenotype (Liu et al. 2002; Kumagai et al. 1995).

Then tobacco total proteins were extracted with an extraction buffer consisting of 50 mM Tris–HCl (pH 7.8), 50 mM NaCl, 10 % (v/v) glycerol, 0.1 % (v/v) Tween-20, 0.15 % (v/v) 2-mercaptoethanol. Gene silenced plants were tested by immunoblotting and were chose for stomatal aperture assay. Stomatal aperture was assayed with small pieces of tobacco leaves essentially as previously described for the assays in Arabidopsis (Shen et al. 2006; Wu et al. 2009).

### Drought treatment

For drought tolerance experiment, plants were grown on soil until they were 3-weeks old when plantlets reached the stage of five to six fully expanded leaves, and drought was imposed by withdrawing irrigation for one-half of the plants until the lethal effects was observed on most of these plants, whereas the other half were grown under a standard irrigation regime as a control.

### Phenotypic analysis

Phenotypic analysis was done essentially as previously described (Shen et al. 2006; Wu et al. 2009; Shang et al. 2010). Briefly, for germination assay, approximately 100 seeds were planted on MS medium (Sigma, St. Louis, MO, USA; product#, M5524; full-strength MS) that contained 3 % sucrose and 0.8 % agar (pH 5.9) and was supplemented with or without (±)-ABA. The seeds were incubated at 4 °C for 3 days, and then placed at 20 °C under light conditions, and germination (emergence of radicals) was scored at the indicated times. Seedling growth was assessed by directly planting the seeds in the ABA-containing MS-medium to investigate the response of seedling growth to ABA after germination. For stomatal aperture assays, 3-week old leaves for *Arabidopsis*, and 5-week old leaves for tobacco were used. To observe ABA-induced stomatal closure, leaves were floated in the buffer containing 50 mM KCl and 10 mM Mes-Tris (pH 6.15) under a halogen cold-light source (Colo-Parmer) at 200 μmol m^−2^ s^−1^ for 2.5 h followed by addition of different concentrations of (±)-ABA. Apertures were recorded on epidermal strips after 2.5 h of further incubation to estimate ABA-induced closure. To study ABA-inhibited stomatal opening, leaves were floated on the same buffer in the dark for 2.5 h before they were transferred to the cold-light for 2.5 h in the presence of ABA, and then apertures were determined.

## Electronic supplementary material

Below is the link to the electronic supplementary material.

Supplementary material 1 (PDF 811 kb)
