# Supplementary material for: Roles of the different components of magnesium chelatase in abscisic acid signal transduction
Source: Plant Mol Biol. 2012 Sep 27;80(4):519–37. doi: 10.1007/s11103-012-9965-3 (PMC3472068; doi:10.1007/s11103-012-9965-3)
Supplement: Supplementary file 1 — Supplementary material 1 (PDF 811 kb) [file 11103_2012_9965_MOESM1_ESM.pdf]

Supplementary data

Du S.Y. *et al.*, Roles of the different components of Mg-chelatase in abscisic acid signal transduction.

(a)

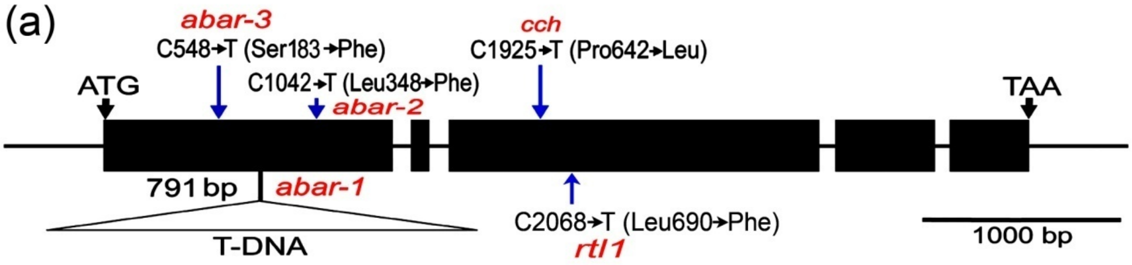

The map of the *abar* mutant alleles is shown above.

(b) **Summary of the ABA-related phenotypes of the mutant alleles**

Mutant allele	Seed germination	Early seedling growth	Stomatal movement
abar-1 (lethal)	----	---	---
abar-2	ABA insensitive	Weakly ABA insensitive	Wild-type
abar-3	ABA hypersensitive	ABA insensitive	Wild-type
cch	Weakly ABA insensitive	Weakly ABA insensitive	Strongly ABA insensitive
rtl1	Weakly ABA insensitive	Wild-type	Strongly ABA insensitive

Supplementary Fig. 1 The identified mutant alleles of *CHLH/ABAR* gene and summary of their ABA-related phenotypes. (a) Diagram showing the locations of the *abar-1*, *abar-2*, *abar-3*, *cch* and *rtl1* mutations in the CHLH/ABAR genomic DNA (modified from Wu et al., 2009). (b) Summary of the ABA-related phenotypes of these mutant alleles.

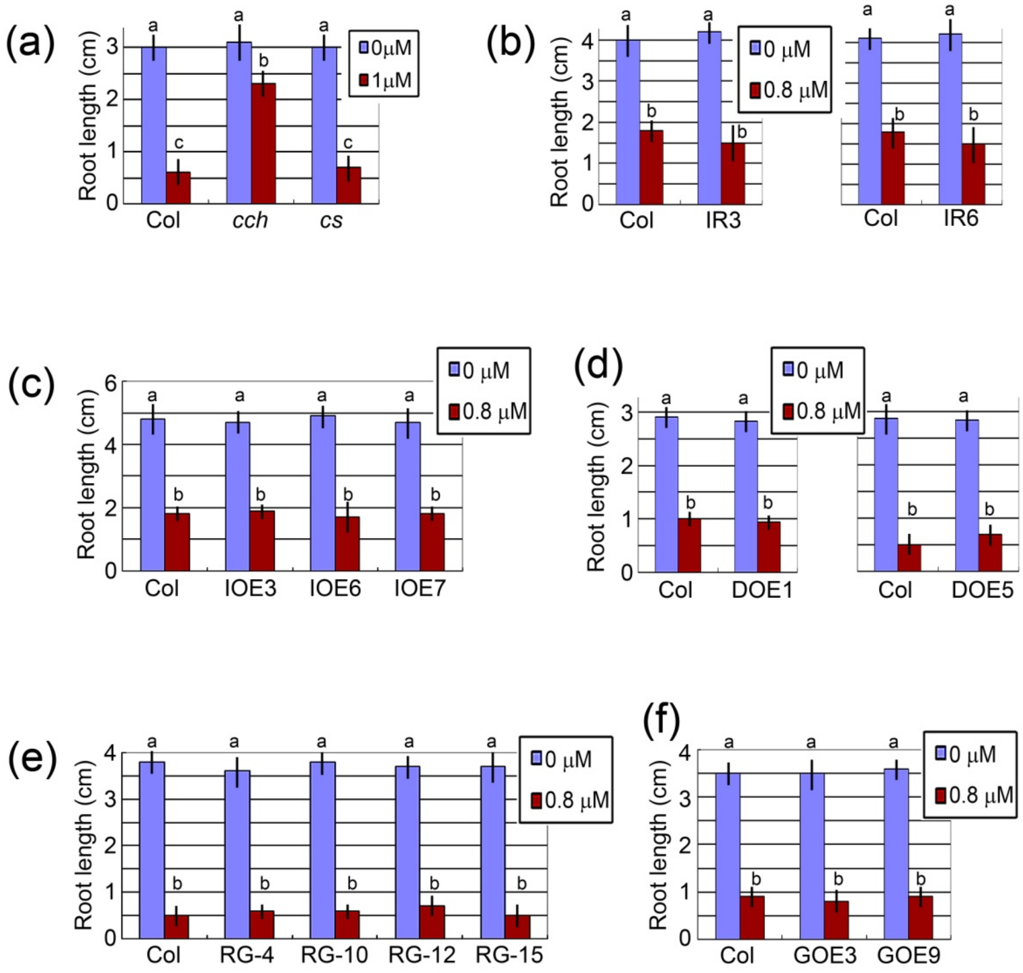

Supplementary Fig. 2 Root length of *cch*, *cs* mutants and different transgenic lines in ABA-containing medium. (a) The data for *cch* and *cs* mutants. For corresponding photos of seedling growth, see Fig. 4c. (b) The data for the *CHLI*-RNAi-transgenic lines IR3 and IR6. For corresponding photos of seedling growth, see Fig. 4d. (c) The data for the *CHLI1*-overexpression transgenic lines IOE3, IOE6 and IOE7. For corresponding photos of seedling growth, see Fig. 4e. (d) The data for the *CHLD*-overexpression transgenic lines DOE1 and DOE5. For corresponding photos of seedling growth, see Fig. 6c. (e) The data for the *GUN4*-RNAi-transgenic lines RG-4, RG-10, RG12 and RG15. For corresponding photos of seedling growth, see Fig. 7c. (f) The data for the *GUN4*-overexpression transgenic lines GOE3 and GOE9. For corresponding photos of seedling growth, see Fig. 7d.

Values are the means ± SE from three independent experiments and different letters indicate significant differences at $P < 0.05$ (Duncan's multiple range test). The experimental conditions are the same as described in the corresponding figures where the photos of the seedlings growth are presented (Fig. 4, 6, or 7).

```
Arabidopsis MASLVYSPFTLSTSKAEHLSSLTNSTK..HSFLRKKHRSTKPAKSFFKVKSAVSGNGLFTQTNPEVRRIVPIKRDNVPT......VKIVY     82
Tobacco     MASLVSSPFTLPNSKVEHLSSISQKHYFLHSFLPKKINPTY.SKSPKKFQCNAIGNGLFTQTTQEVRRIVPENTQGLAT......VKIVY     83
Barley      MSSLVSAPFATATGAQKKARGPRPAPLHSFLLTGR.......RGRRAT..IRCAVAGNGLFTQTNPDVRRVVPAE.....RGLPRVKVVY     76
Rice        MSSLVSTPFTTATGVQKKLGAPV..PLHSFLLSRRQPAAGAGRGRAAAAAIRCAVAGNGLFTQTKPEVRRVVPPEGDASRRGVPRVKVVY     88
Soybean     MASLVSSPFTLPSSKPDQLHSLAQKHLYLHSFLPKKANYNGSSKSSLRVKCAVIGNGLFTQTTQEVRRIVPENDQNLPT......VKIVY     84

Arabidopsis VVLEAQYQSSLSEAVQSLNKTSRFAS.YEVVGYLVEELRDKNTYNNFCEDLKDANIFIGSLIFVEELAIKVKDAVEKERDRMDAVLVFPS    171
Tobacco     VVLEAQYQSSLTAAVQTLNKNGQFAS.FEVVGYLVEELRDENTYKMFCKDLEDANVFIGSLIFVEELALKVKSAVEKERDRLDAVLVFPS    172
Barley      VVLEAQYQSSVTAAVMQLNADPRRAAEFEVVGYLVEELRDADTYAAFCDDVAAANVFIGSLIFVEELALKVRDAVAKHRDRMDAVLVFPS    166
Rice        VVLEAQYQSSVTAAVRELNADPRRAAGFEVVGYLVEELRDEETYKTFCADLADANVFIGSLIFVEELALKVKDAVEKERDRMDAVLVFPS    178
Soybean     VVLEAQYQSSITAAVIALNSKRKHAS.FEVVGYLVEELRDAATYKTFCKDLEDANIFIGSLIFVEELALKIKAAVEKERDRLDAVLVFPS    173

Arabidopsis MPEVMRLNKLGSFSMSQLGQSKSPFFQLFKRKKQGSAGFADSMLKLVRTLPKVLKYLPSDKAQDARLYILSLQFWLGGSPDNLQNFVKMI    261
Tobacco     MPEVMRLNKLGSFSMSQLGQSKSPFFELFKKKKPSSAGFSDQMLKLVRTLPKVLKYLPSDKAQDARLYILSLQFWLGGSPDNLVNFLKMI    262
Barley      MPEVMRLNKLGSFSMAQLGQSKSPFFQLFKRNKKDSSGFADSMLKLVRTLPKVLKYLPSDKAQDARLYILSLQFWLGGSPDNLQNFLKMI    256
Rice        MPEVMRLNKLGSFSMSQLGQSKSPFFQLFKR.KKNSGGFADSMLKLVRTLPKVLKYLPSDKAQDARLYILSLQFWLGGSPDNLQNFLKMI    267
Soybean     MPEVMRLNKLGSFSMSQLGQSKSPFFQLFKRKKPQSAGFADSMLKLVRTLPKVLKYLPSDKAQDARLYILSLQFWLGGSPDNLQNFLKMI    263

Arabidopsis SGSYVPALKGVKIEYSDPVLFLDTGIWHPLAPTMYDDVKEYWNWYDTRRDTNDSLKRKDATVVGLVLQRSHIVTGDDSHYVAVIMELEAR    351
Tobacco     SGSYVPALKGMKIDYSDPVLYLDNGIWHPLAPCMYDDVKEYLNWYATRRDTNEKLKSSNAPVVGLVLQRSHIVTCDESHYVAVIMELEAK    352
Barley      AVSYVPALKGADIRYNDPVLFLDTGIWHPLAPTMYDDVKEYLNWYGTRRDANDRLKNPEAPVIGLVLQRSHIVTGDDGHYVAVIMELEAR    346
Rice        AVSYVPALKGADIKYDDPVLFLDAGIWHPLAPTMYDDVKEYLNWYGTRRDTNDKLKDPNAPVIGLVLQRSHIVTGDDGHYVAVIMELEAK    357
Soybean     SGSYIPALKGTKIEYSEPVLYLDVGIWHPLAPCMYDDVKEYLNWYGTRRDANEKLKSPNAPVIGLVLQRSHIVTGDDGHYVAVIMELEAR    353

Arabidopsis GAKVVPIFAGGLDFSGPVEKYFVDPVSKQPIVNSAVSLTGFALVGGPARQDHPRAIEALKKLDVPYLVAVPLVFQTTEEWLNSTLGLHPI    441
Tobacco     GAKVIPIFAGGLDFSRPIERYFIDPITKKPFVNSVISLSGFALVGGPARQDHPRAIEALMKLDVPYIVALPLVFQTTEEWLNSTLGLHPI    442
Barley      GAKVIPIFAGGLDFSGPIERYLVDPITKKPFVNAVVSLTGFALVGGPARQDHPKAIASLMKLDVPYIVALPLVFQTTEEWLNSTLGLHPI    436
Rice        GAKVIPIFAGGLDFSGPTQRYLVDPITGKPFVNAVVSLTGFALVGGPARQDHPKAIAALQKLDVPYIVALPLVFQTTEEWLNSTLGLHPI    447
Soybean     GAKVIPIFAGGLDFSGPVEKFFIDPITKKPFVNSVVSLTGFALVGGPARQDHPRAVEALMKLDVPYIVALPLVFQTTEEWLNSTLGLHPI    443

Arabidopsis QVALQVALPELDGAMEPIVFAGRDPRTGK.....SHALHKRVEQLCIRAIRWGELKRKTKAEKKLAITVFSFPPDKGNVGTAAYLNVFAS    526
Tobacco     QVALQVALPELDGGMEPIVFAGRDPRTGK.....SHALHKRVEQLCTRAIKWGELKRKTKAEKRLAITVFSFPPDKGNVGTAAYLNVFAS    527
Barley      QVALQVALPELDGGMEPIVFAGRDPRSGKPLLRKSHALHKRVEQLCTRAIRWAELKRKTKMDKKLAITVFSFPPDKGNVGTAAYLNVFSS    526
Rice        QVALQVALPELDGGMEPIVFAGRDPRTGK.....SHALHKRVEQLCTRAIRWAELKRKTKEEKKLAITVFSFPPDKGNVGTAAYLNVFNS    532
Soybean     QVALQVALPELDGGMEPIVFAGRDPKTGK.....SHALHKRVEQLCIRAIRWAELKRKSKEEKKLAITVFSFPPDKGNVGTAAYLNVFAS    528

Arabidopsis IFSVLRDLKRDGYNVEGLPENAETLIEEIIHDKEAQFSSPNLNVAYKMGVREYQDLTPYANALEENWGKPPGNLNSDGENLLVYGKAYGN    616
Tobacco     IYSVLKDLKKDGYNVEGLPETSAQLIEEVIHDKEAQFSSPNLNIAYKMNVREYQKLTPYATALEENWGKAPGNLNSDGENLLVYGKQYGN    617
Barley      IYSVLRDLKKDGYNVEGLPETPEELIEEVIHDKEAQFNSPNLNVVYRMNVREYQALTPYANMLEENWGKPPGHLNSDGENLLVYGKQYGN    616
Rice        IYSVLQDLKKDGYNVEGLPDTAEALIEEVIHDKEAQFNSPNLNVAYRMNVREYQSLTSYASLLEENWGKPPGNLNSDGENLLVYGKQYGN    622
Soybean     IYSVMKELKKDGYNVDGLPETSEALIEDVLHDKEAQFSSPNLNIAYKMNVREYQNLTPYATALEENWGKPPGNLNADGENLLVYGKQYGN    618

Arabidopsis VFIGVQPTFGYEGDPMRLLFSKSASPHHGFAAYYSYVEKIFKADAVLHFGTHGSLEFMPGKQVGMSDACFPDSLIGNIPNVYYYAANNPS    706
Tobacco     VFIGVQPTFGYEGDPMRLLFSKSASPHHGFAAYYSFVEKIFKADAVLHFGTHGSLEFMPGKQVGMSDASFPDSLIGNIPNVYYYAANNPS    707
Barley      IFIGVQPTFGYEGDPMRLLFSKSASPHHGFAAYYTFVEKIFKADAVLHFGTHGSLEFMPGKQVGMSDACFPDSLIGNIPNIYYYAANNPS    706
Rice        VFIGVQPTFGYEGDPMRLLFSKSASPHHGFAAYYTFVEKIFQADAVLHFGTHGSLEFMPGKQVGMSDACYPDSLIGNIPNIYYYAANNPS    712
Soybean     VFIGVQPTFGYEGDPMRLLFSKSASPHHGFAAYYSFVEKIFKADAVLHFGTHGSLEFMPGKQVGMSDVCYPDSLIGNIPNVYYYAANNPS    708

Arabidopsis EATIAKRRSYANTISYLTPPAENAGLYKGLKQLSELISSYQSLKDTGRGPQIVSSIISTAKQCNLDKDVDLPDEGLELSPKDRDSVVGKV    796
Tobacco     EATIAKRRSYANTISYLTPPAENAGLYKGLKQLSELISSYQSLKDSGRGQQIVNSIISTARQCNLDKDVDLPEEGEEISAKERDLVVGKV    797
Barley      EATVAKRRSYANTISYLTPPAENAGLYKGLKQLSELIASYQSLKDTGRGNQIVSSIISTAKQCNLDKDVALPDEGEELPANERDLVVGKV    796
Rice        EATVAKRRSYANTISYLTPPAENAGLYKGLKQLSELISSYQSLKDTGRGPQIVSSIISTAKQCNLDKDVPLPEEGVELPPNERDLIVGKV    802
Soybean     EATIAKRRSYANTISYLTPPAENAGLYKGLKQLSELISSYQSLKDTGRGAQIVSSIISTAKQCNLDKDVTLPDEGEEIPPKERDLVVGQV    798

Arabidopsis YSKIMEIESRLLPCGLHVIGEPPSAMEAVATLVNIAALDRPEDEISALPSILAECVGREIEDVYRGSDKGILSDVELLKEITDASRGAVS    886
Tobacco     YSKIMEIESRLLPCGLHIIGEPPTAMEAVATLVNIATLDRPEEGISALPSILAATVGRSIEEIYRGNDQGILRDVELLRQITEASRGAIS    887
Barley      YGKLMEIESRLLPCGLHVIGEPPTAVEAVATLVNIAALDRPEENIFSLPGILAATVGRTIEDVYRGSDKGILADVELLKQITEASRGAVG    886
Rice        YAKIMEIESRLLPCGLHVIGEPPSAIEAVATLVNIASLDRPEDEIYSLPNILAQTVGRNIEDVYRGSDKGILADVELLRQITEASRGAIT    892
Soybean     YSKIMEIESRLLPCGLHIIGEPPSALEAVATLVNIAALDRPEDGISSLPSILADTVGRDIEDVYRGSNKGILKDVELLRQITEASRGAIT    888

Arabidopsis AFVEKTTNSKGQVVDVSDKLTSLLGFGINEPWVEYLSNTKFYRANRDKLRTVFGFLGECLKLVVMDNELGSLMQALEGKYVEPGPGGDPI    976
Tobacco     AFVERTTNNKGQVVNVNDKLTSILGFGINEPWIQYLSNTQFYRADRDKLRVLFQFLGECLKLIVANNEVGSLKQALEGKYVEPGPGGDPI    977
Barley      AFVEKSTNSKGQVVDVTSKLSSILGFGLSEPWVEYLSQTKFIRADRDKLRTLFGFLGECLKLIVADNELGALKTALEGSYVEPGPGGDPI    976
Rice        TFVERTTNNKGQVVDVTNKLSTMLGFGLSEPWVQHLSKTKFIRADREKLRTLFTFLGECLKLIVADNELGSLKLALEGSYVEPGPGGDPI    982
Soybean     AFVERTTNNKGQVVDVADKLSSILGFGINEPWIQYLSNTKFYRADREKLRTLFVFLGECLKLIVADNEVGSLKQALEGKYVEPGPGGDPI    978

Arabidopsis RNPKVLPTGKNIHALDPQAIPTTAAMASAKIVVERLVERQKLENEGKYPETIALVLWGTDNIKTYGESLGQVLWMIGVRPIADTFGRVNR   1066
Tobacco     RNPKVLPTGKNIHALDPQAIPTIAAVQSAKIVVERLLERQKADNGGKYPETVALVLWGTDNIKTYGESLAQVMWMIGVRPVTDSLGRVNR   1067
Barley      RNPKVLPTGKNIHALDPQSIPTAAAMKSAKIVVERLLERQKADNGGKYPETIALVLWGTDNIKTYGESLAQVMWMLGVEPVTDGLGRVNR   1066
Rice        RNPKVLPTGKNIHALDPQAIPTTAALKSAKIIVDRLLERQKVDNGGKYPETIALVLWGTDNIKTYGESLAQVLWMIGVRPVADTFGRVNR   1072
Soybean     RNPKVLPTGKNIHALDPQSIPTTAAMQSAKIVVDRLIERQKAENGGKYPETIALVLWGTDNIKTYGESLAQVLWMIGVEPVADTFGRVNR   1068

Arabidopsis VEPVSLEELGRPRIDVVVNCSGVFRDLFINQMNLLDRAIKMVAELDEPVEQNFVRKHALEQAEALGIDIREAATRVFSNASGSYSANISL   1156
Tobacco     VEPVSLEELGRPRVDVVVNCSGVFRDLFINQMNLLDRAVKMVAELDEPEDQNYVRKHALEQAKTLGVDVREAATRIFSNASGSYSSNINL   1157
Barley      VEPVSIEELGRPRIDVVVNCSGVFRDLFINQMNLLDRAVKMVAELEEPIEMNYVRKHAMEQAEELGVSVREAATRIFSNASGSYSSNVNL   1156
Rice        VEPVSLEELGRPRIDVVINCSGVFRDLFINQMNLLDRAVKMVAELDEPEEMNYVRKHAQEQARELGVSLREAATRVFSNASGSYSSNVNL   1162
Soybean     VEPVSLEELGRPRIDVVVNCSGVFRDLFINQMNLLDRAVKMVAELDEPAEQNYVKKHASEQAQALGVEVREAATRIFSNASGSYSSNINL   1158

Arabidopsis AVENSSWNDEKQLQDMYLSRKSFAFDSDAPGAGMAEKKQVFEMALSTAEVTFQNLDSSEISLTDVSHYFDSDPTNLVQSLRKDKKKPSSY   1246
Tobacco     AVENSTWNDEKQLQDMYLSRKSFAFDCDAPGVGMTEKRKVFEMALSTADATFQNLDSSEISFTDVSHYFDSDPTNLVQNLRKDGKKPSAY   1247
Barley      AVENASWTDEKQLQDMYLSRKSFAFDSDAPGVGMLEKRKTFELALATADATFQNLDSSEISLTDVSHYFDSDPTKLVQGLRKDGRAPSSY   1246
Rice        AVENASWTDEKQLQDMYLSRKSFAFDCDAPGAGMREQRKTFELALATADATFQNLDSSEISLTDVSHYFDSDPTKLVQGLRKDGRAPSSY   1252
Soybean     AVENSSWNDEKQLQDMYLSRKSFAFDSDAPGAGMTEKRKVFEMALSTADATFQNLDSSEISLTDVSHYFDSDPTNLVQNLRKDGKKPSAY   1248

Arabidopsis IADTTTANAQVRTLSETVRLDARTKLLNPKWYEGMMSSGYEGVREIEKRLSNTVGWSATSGQVDNWVYEEANSTFIQDEEMLNRLMNTNP   1336
Tobacco     IADTTTANAQVRTLSETVRLDARTKLLNPKWYEGMLSTGYEGVREIEKRLTNTVGWSATSGQVDNWVDEEANTTFIQDQEMLNRLMNTNP   1337
Barley      IADTTTANAQVRTLSETVRLDARTKLLNPRWYEGMMKSGYEGVREIEKRLTNTVGWSATSGQVDNWVYEEANTTFIEDEEMRKRLMDTNP   1336
Rice        IADTTTANAQVRTLSETVRLDARTKLLNPKWYEGMMKSGYEGVREIEKRLTNTVGWSATSGQVDNWVYEEANATFIEDEAMRKRLMDTNP   1342
Soybean     IADTTTANAQVRTLSETVRLDARTKLLNPKWYEGMLSTGYEGVREIEKRLTNTVGWSATSGQVDNWVYEEANTTFIQDEQMLNKLMSTNP   1338

Arabidopsis NSFRKMLQTFLEANGRGYWDTSAENIEKLKELYSQVEDKIEGID                                                1380
Tobacco     NSFRKLLQTFLEANGRGYWETSAENIEKLKQLYSEVEDKIEGID                                                1381
Barley      NSFRKLLQTFLEANGRGYWETSEDNLERLRELYSEVEDKIEGID                                                1380
Rice        NSFRKLVQTFLEASGRGYWETSEENLEKLRELYSEVEDKIEGID                                                1386
Soybean     NSFRKLVQTFLEANGRGYWETSEDNIEKLRQLYSEVEDKIEGID                                                1382
```

Supplementary Fig. 3 Alignment of the Amino Acid Sequence of Five Mg-Chelatase H Subunits (Identity = 89.30%).

```
Arabidopsis MAMTPVASSSPVSTCRLFRCNLLPDLLPKPLFLSLPKRNRIASCRFTVRA...SA.NATVESPNGVPASTSDTDTETDTT     76
Tobacco     ..MGFCSTSTLPQTSLSNSQSSTFFTYLKPCPILSSTYLRPKRLKFRLRI...SA.TATIDSPNGAVAVVEPEKQPEKIS     74
Barley      ....MAAMATALSTSLPH.LPPRRLPSHPVAALSLAPRGFRRREAPARLAAVASA.SEVLDSTNGAAPAPTSPAPSGQQK     74
Rice        ....MAMATTALSASLPRLLPPRRRRFPTPSSSSPSAASTSTSRVVRLRAAAASAPSEVLDSTNGAIPSGKGGG..GQQ.     73
Soybean     .MGFALAYTASGCCSNLQFQSLLFAAASLRSKPCLSLCNSTYRPKRILQR...SP.IVGAQSENGALVTSEKPDTNY...     72

Arabidopsis SYGRQFFPLAAVVGQEGIKTALLLGAVDREIGGIAISGRRGTAKTVMARGLHEILPPIEVVVGSISNADPACPDEWEDDL    156
Tobacco     F.GRQYFPLAAVIGQDAIKTALLLGAIDREIGGIAICGKRGTAKTLMARGLHAILPPIEVVVGSMANADPNCPDEWEDGL    153
Barley      Y.GREYFPLAAVVGQDAIKTSLLLGAIDREVGGIAISGKRGTAKTVMARGLHAMLPPIEVVVGSIANADPNIPEEWEDHL    153
Rice        Y.GREYFPLAAVVGQDAIKTALLLGAIDREIGGIAISGKRGTAKTVMARGLHAMLPPIEVVVGSIANADPNYPEEWEEGL    152
Soybean     ..GRQYFPLAAVVGQDSIKTALLLGAIDPGVGGIAISGKRGTAKTVMARGLHAILPPIEVVEGSIANADPTCPEEWEDGL    150

Arabidopsis DERIEYNADNTIKTEIVKSPFIQIPLGVTEDRLIGSVDVEESVKRGTTVFQPGLLAEAHRGVLYVDEINLLDEGISNLLL    236
Tobacco     ADRAEYGSDGNIKTQIVKSPFVQIPLGVTEDRLIGSVDVEESVKSGTTVFQPGLLAEAHRGVLYVDEINLLDEGISNLLL    233
Barley      ADQVQYDADGNVKCEIVKAPFVQIPLGVTEDRLIGSVDVEQSVRSGTTVFQPGLLAEAHRGVLYVDEINLLDDGISNLLL    233
Rice        ANQVQYDADGNLKTEIIKTPFVQIPLGITEDRLIGSVDVEASVKSGTTVFQPGLLAEAHRGVLYVDEINLLDEGVSNLLL    232
Soybean     TECLEYDSTGNIKTRIIKSPFVQIPLGVTEDRLIGSVDVEESVKTGTTVFQPGLLAEAHRGVLYVDEINLLDEGISNLLL    230

Arabidopsis NVLTDGVNIVEREGISFRHPCKPLLIATYNPEEGAVREHLLDRVAINLSADLPMSFEDRVAAVGIATQFQERCNEVFRMV    316
Tobacco     NVLTEGVNIVEREGISFRHPCKPLLIATYNPEEGAVREHLLDRIAINLSADLPMSFDDRVAAVDIATRFQECSNEVFKMV    313
Barley      NVLTEGVNIVEREGISFRHPCKPLLIATYNPEEGSVREHLLDRIAINLSADLPLSFDDRVAAVNIATQFQESSKDVFKMV    313
Rice        NVLTEGVNIVEREGISFRHPCKPLLIATYNPEEGSVREHLLDRIAINLSADLPMSFDDRVAAVDIATQFQESSKEVFKMV    312
Soybean     NVLTEGVNTVEREGISFKHPCRPLLIATYNPEEGAVREHLLDRIAINLSADLPMSFENRVAAVGIATEFQENSSQVFEMV    310

Arabidopsis NEETETAKTQIILAREYLKDVKISREQLKYLVLEAVRGGVQGHRAELYAARVAKCLAAIEGREKVTIDDLRKAVELVILP    396
Tobacco     DEETDSAKTQIILAREYLKDVTISRDQLKYLVMEAIRGGCQGHRAELYAARVAKCLAAIDGREKVGVDELKKAVELVILP    393
Barley      EEETEVAKTQIILAREYLKDVAISTEQLKYLVMEAIRGGCQGHRAELYAARVAKCLAAMEGREKVFAEDLKKAVELVILP    393
Rice        EEETEVAKTQIILAREYLKDVAISTEQLKYLVMEAIRGGCQGHRAELYAARVAKCLAAMEGREK.........VELVILP    383
Soybean     EEETDNAKTQIILAREYLKDVTLNREQLKYLVIEALRGGCQGHRAELFAARVAKCLAALEGREKVYVDDLKKAVELVILP    390

Arabidopsis RSSLDETPPEQQN.QPPPPPPPQN........SESGEEENEEEQEEEEDESNEENENEQQQDQIPEEFIFDAEGGLVD    467
Tobacco     RSTIVENPPDQQNQQPPPPPPPQN........QDSSEEQNEEEEKEEEDQEDEKDRENEQQQPQVPDEFIFDAEGGLVD    465
Barley      RSIISDNPQEQQN.QPPPPPPPPPQNQDNAEDQDEKEED...EEKDEEEKEDDDE.ENEKQDDQIPEEFIFDAEGGLVD    468
Rice        RSILSDNPQEQQDQQPPPPPPPPPQDQDSQEDQDEDE.........EEDQEDDDE.ENEQQDQQIPEEFIFDAEGGIVD    453
Soybean     RSIVTENPPDQQN.QPPPPPPPQN........QESGEEQNEEEE.....QEDDKDEENEQQQEQLPEEFIFDAEGGLVD    456

Arabidopsis EKLLFFAQQAQKRRGKAGRAKNVIFSEDRGRYIKPMLPKGPVKRLAVDATLRAAAPYQKLRREKDISGTRKVFVEKTDMR    547
Tobacco     EKLLFFAQQAQRRKGKAGRAKKVIFSEDRGRYIKPMLPKGPVKRLAVDATLRAAAPYQKLRRAKDIQKTRKVYVEKTDMR    545
Barley      DKLLFFAQQAQRKKGKAGRAKNVIFSEDRGRYIKPMLPKGPVRRLAVDATLRAAAPYQKLRREKSLDKTRKVFVEKTDMR    548
Rice        EKLLFFAQQAQRRRGKAGRAKNLIFSSDRGRYIGSMLPKGPIRRLAVDATLRAAAPYQKLRREKDRDKTRKVFVEKTDMR    533
Soybean     EKLLFFAQQAQRRRGRAGRAKNVIFSEDRGRYIKPMLPKGPVKRLAVDATLRAAAPYQKLRRAKDSGNNRKVFVEKTDMR    536

Arabidopsis AKRMARKAGALVIFVVDASGSMALNRMQNAKGAALKLLAESYTSRDQVSIIPFRGDAAEVLLPPSRSIAMARNRLERLPC    627
Tobacco     AKRMARKAGALVIFVVDASGSMALNRMQNAKGAALKLLAESYTSRDQVCIIPFRGDAAEVLLPPSRSISMARNRLERLPC    625
Barley      AKRMARKAGALVIFVVDASGSMALNRMQNAKGAALKLLAESYTSRDQVAIIPFRGDYAEVLLPPSRSIAMARKRLEKLPC    628
Rice        AKRMARKAGALVIFVVDASGSMALNRMQNAKGAALKLLAESYTSRDQVSIIPFRGDFAEVLLPPSRSIAMARNRLEKLPC    613
Soybean     AKRMARKAGALVIFVVDASGSMALNRMQNAKGAALKLLAESYTSRDQVSIIPFRGDAAEVLLPPSRSISMARKRLERLPC    616

Arabidopsis GGGSPLAHGLTTAVRVGLNAEKSGDVGRIMIVAITDGRANITLKRSTDPESI.APDAPRPTSKELKDEILEVAGKIYKAG    706
Tobacco     GGGSPLAHGLTTAVRVGMNAEKSGDVGRIMIVAITDGRANISLKRSTDPEAE.AS.DAPRPSSQELKDEILEVAGKIYKT    703
Barley      GGGSPLAHGLSTAVRVGLNAEKSGDVGRIMIVAITDGRANVSLKKSNDPEAAAS.DAPRPSTQELKDEILDVSAKIFKA    707
Rice        GGGSPLAHGLSTAVRVGLNAEKSGDVGRIMIVAITDGRANVSLKKSTDPEAT..S.DAPRPSSQELKDEILEVAGKIYKA    690
Soybean     GGGSPLAHGLTTAVRVGLNAEKSGDVGRIMIVAITDGRANISLKRSTDPEAA.AATDAPKPSAQELKDEILEVAGKIYKA    695

Arabidopsis MSLLVIDTENKFVSTGFAKEIARVAQGKYYYLPNASDAVISATTRDALSDLKNS                                 760
Tobacco     GMSLLVIDTENKFVSTGFAKEIARVAQGKYYYLPNASDAVISAATKDALSALKE                                 757
Barley      GMSLLVIDTENKFVSTGFAKEIARVAQGKYYYLPNASDAVISAATKTALADLKS                                 761
Rice        GISLLVIDTENKFVSTGFAKEIARVAQGKYYYLPNASDAVISAATKTALSDLKS                                 744
Soybean     GMSLLVIDTENKFVSTGFAKEIARVAQGKYYYLPNASDAVISSATKEALSALKS                                 749
```

Supplementary Fig. 4 Alignment of the Amino Acid Sequence of Five Mg-Chelatase D Subunits (Identity = 82.94%).

```
Tabacco     ..MASLLGTSSSAAAAILASTPLSSRSCKPAVFSLFP.SSGQSQGRKFYGGIRVPVKKGRSQFHVAISNVATEINLLKNR   77
Arabidopsis ..MASLLGTSSSAIWASPSLSSPSSKPSSSPICFRPG.KLFGSKLNAGIQIRPKKNRSRYHV.SVM..NVATEINSTEQV   74
Barley      MAMASPFSPASAA.AASPALFAVSTSRP......LSLTTAATAAVSARAPSRTRSGLRRGRFAVC...NVAAPSAAEQET   70
Rice        ..MASAFSPATAAPAASPALFSASTSRP......LSL.TAAAAAVSARIPSRRGF..RRGRFTVC...NVAAPSATQQEA   66
Soybean     ..MASALGTSSIAVLPSRYF...SSSSSKPSIHTLSL.TSGQNYGRKFYGGIGIHGIKGRAQLSVT..NVATEVNSVEQA   72

Tabacco     V.RNL.LEESQRPVYPFAAIVGQDEMKLCLLLNVIDPKIGGVMIMGDRGTGKSTTVRSLVDLLPEIKVISGDPFNSDPDD  155
Arabidopsis V.GKFDSKKSARPVYPFAAIVGQDEMKLCLLLNVIDPKIGGVMIMGDRGTGKSTTVRSLVDLLPEINVVAGDPYNSDPID  153
Barley      KPAAA.AKESQRPVYPFPAIVGQDEMKLCLLLNVIDPKIGGVMIMGDRGTGKSTTVRSLVDLLPDISVVVGDPFNSDPYD  149
Rice        K.AAG.AKESQRPVYPFAAIVGQDEMKLCLLLNVIDPKIGGVMIMGDRGTGKSTTVRSLVDLLPDIRVVVGDPFNSDPDD  144
Soybean     Q.SIA.SKESQRPVYPFSAIVGQDEMKLCLLLNVIDPKIGGVMIMGDRGTGKSTTVRSLVDLLPEIKVVAGDPYNSDPQD  150

Tabacco     QEVMSAEVRDKLRSGQQLPISRTKINMVDLPLGATEDRVCGTIDIEKALTEGVKAFEPGLLAKANRGILYVDEVNLLDDH  235
Arabidopsis PEFMGVEVRERVEKGEQVPVIATKINMVDLPLGATEDRVCGTIDIEKALTEGVKAFEPGLLAKANRGILYVDEVNLLDDH  233
Barley      PEVMGPEVRDRLLKGESLPVTTTKITMVDLPLGATEDRVCGTIDIDKALTEGVKAFEPGLLAKANRGILYVDEVNLLDDH  229
Rice        PEVMGPEVRERVLEGEKLPVVTAKITMVDLPLGATEDRVCGTIDIEKALTDGVKAFEPGLLAKANRGILYVDEVNLLDDH  224
Soybean     PEFMGVEVRERVLQGEELSVVLTKINMVDLPLGATEDRVCGTIDIEKALTEGVKAFEPGLLAKANRGILYVDEVNLLDDH  230

Tabacco     LVDVLLDSAASGWNTVEREGISISHPARFILIGSGNPEEGELRPQLLDRFGMHAQVGTVRDAELRVKIVEERARFDKNPK  315
Arabidopsis LVDVLLDSAASGWNTVEREGISISHPARFILIGSGNPEEGELRPQLLDRFGMHAQVGTVRDADLRVKIVEERARFDSNPK  313
Barley      LVDVLLDSAASGWNTVEREGISISHPARFILIGSGNPEEGELRPQLLDRFGMHAQVGTVRDAELRVKIVEERARFDRDPK  309
Rice        LVDVLLDSAASGWNTVEREGISISHPARFILIGSGNPEEGELRPQLLDRFGMHAQVGTVRDAELRVKIVEERARFDRDPK  304
Soybean     LVDVLLDSAASGWNTVEREGISISHPARFILIGSGNPEEGELRPQLLDRFGMHAQVGTVRDAELRVKIVEERGRFDKNPK  310

Tabacco     EFRESYKAEQEKLQNQIDSARNALSAVTIDHDLRVKISKVCAELNVDGLRGDIVTNRAARALAALKGRDKVTPEDIATVI  395
Arabidopsis DFRDTYKTEQDKLQDQISTARANLSSVQIDRELKVKISRVCSELNVDGLRGDIVTNRAAKALAALKGKDRVTPDDVATVI  393
Barley      TFRQSYLEEQDKLQEQITSARSNLGSVQLDHDLRVKISQVCSELNVDGLRGDIVTNRAAKALAALKGKDVVTVEDISTVI  389
Rice        AFRESYLEEQDKLQQQISSARSNLGAVQIDHDLRVKISKVCAELNVDGLRGDIVTNRAAKALAALKGRDTVTVEDIATVI  384
Soybean     EFRDSYKAEQEKLQQQITSARSVLSSVQIDQDLKVKISKVCAELNVDGLRGDIVTNRAAKALAALKGRDNVSAEDIATVI  390

Tabacco     PNCLRHRLRKDPLESIDSGVLVVEKFYEVF                                                    425
Arabidopsis PNCLRHRLRKDPLESIDSGVLVSEKFAEIF                                                    423
Barley      PTVLRHRLRKDPLESIDSGLLVVEKFYEVF                                                    419
Rice        PNCLRHRLRKDPLESIDSGLLVVEKFYEVF                                                    414
Soybean     PNCLRHRLRKDPLESIDSGLLVTEKFYEVF                                                    420
```

Supplementary Fig. 5 Alignment of the Amino Acid Sequence of Five Mg-Chelatase I Subunits (Identity = 82.74%).

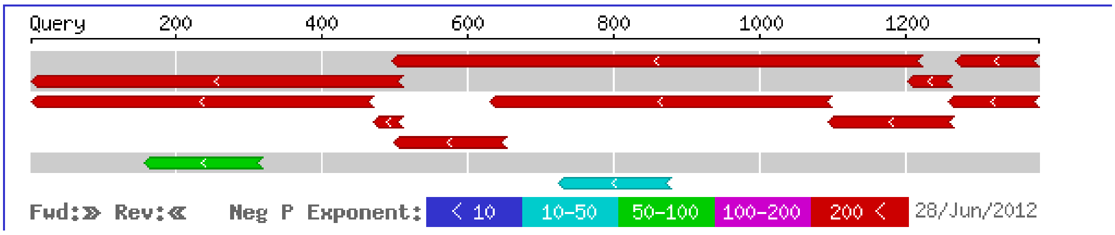

1. Alignment summary of CHLH from *Arabidopsis thaliana* and *Nicotiana benthamiana*

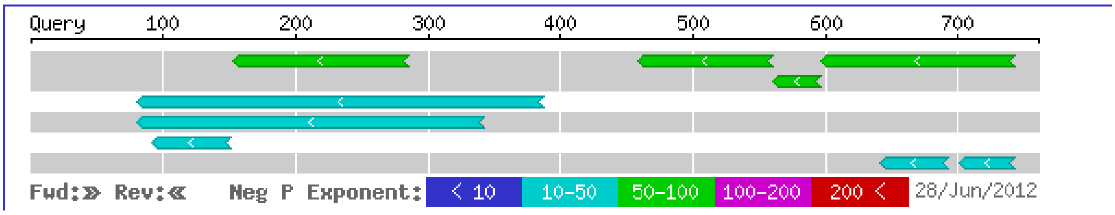

2. Alignment summary of CHLD from *Arabidopsis thaliana* and *Nicotiana benthamiana*

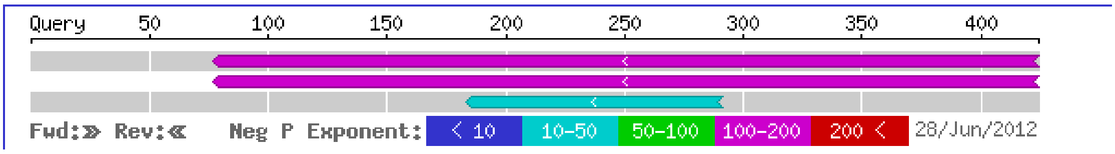

3. Alignment summary of CHLI from *Arabidopsis thaliana* and *Nicotiana benthamiana*

Supplementary Fig. 6 Alignment of the Amino Acid Sequence of Mg-Chelatase H, D and I Subunits of *Arabidopsis thaliana* and *Nicotiana benthamiana*.

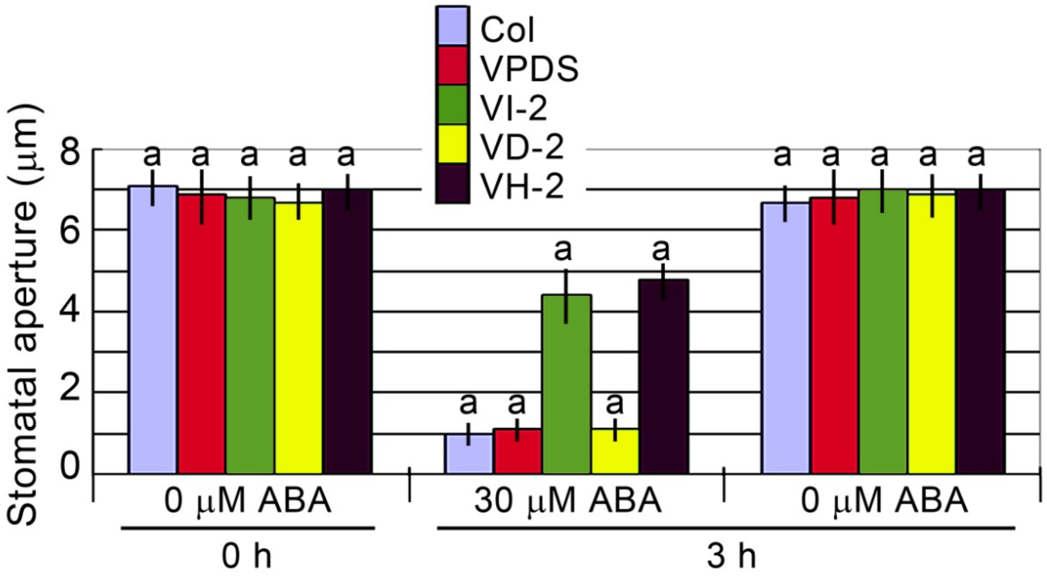

Supplementary Fig. 7 ABA-induced stomatal closure in the VIGS transgenic tobacco leaves. WT, wild-type plants; VPDS, VI-2, VD-2 and VH-2, the same transgenic lines as described in Fig. 5a. Stomatal aperture was assayed in the ABA-free medium (0 μM ABA) and ABA-containing medium (30 μM). Values are the means ± SE from three independent experiments and different letters indicate significant differences at $P < 0.05$ (Duncan's multiple range test) when comparing values within the same ABA concentration. n = 60 apertures per experiment.
